# Room-Temperature Phosphorescent Organic-Doped Inorganic Frameworks Showing Wide-Range and Multicolor Long-Persistent Luminescence

**DOI:** 10.34133/2021/9862327

**Published:** 2021-04-09

**Authors:** Guowei Xiao, Bo Zhou, Xiaoyu Fang, Dongpeng Yan

**Affiliations:** ^1^Beijing Key Laboratory of Energy Conversion and Storage Materials, College of Chemistry Beijing Normal University, Beijing 100875, China; ^2^College of Chemistry, Key Laboratory of Radiopharmaceuticals, Ministry of Education, Beijing Normal University, Beijing 100875, China; ^3^College of Chemistry and Molecular Engineering, Zhengzhou University, Zhengzhou 450001, China

## Abstract

Long-persistent luminescence based on purely inorganic and/or organic compounds has recently attracted much attention in a wide variety of fields including illumination, biological imaging, and information safety. However, simultaneously tuning the static and dynamic afterglow performance still presents a challenge. In this work, we put forward a new route of organic-doped inorganic framework to achieve wide-range and multicolor ultralong room-temperature phosphorescence (RTP). Through a facile hydrothermal method, phosphor (tetrafluoroterephthalic acid (TFTPA)) into the CdCO_3_ (or Zn_2_(OH)_2_CO_3_) host matrix exhibits an excitation-dependent colorful RTP due to the formation of diverse molecular aggregations with multicentral luminescence. The RTP lifetime of the doped organic/inorganic hybrids is greatly enhanced (313 times) compared to the pristine TFTPA. The high RTP quantum yield (43.9%) and good stability guarantee their easy visualization in both ambient and extreme conditions (such as acidic/basic solutions and an oxygen environment). Further codoped inorganic ions (Mn^2+^ and Pb^2+^) afford the hybrid materials with a novel time-resolved tunable afterglow emission, and the excitation-dependent RTP color is highly adjustable from dark blue to red, covering nearly the whole visible spectrum and outperforming the current state-of-the-art RTP materials. Therefore, this work not only describes a combined codoping and multicentral strategy to obtain statically and dynamically tunable long-persistent luminescence but also provides great opportunity for the use of organic-inorganic hybrid materials in multilevel anticounterfeiting and multicolor display applications.

## 1. Introduction

Long-persistent luminescent systems have attracted great attention due to their effective utilization of the excited state energy and long-lived photoemission, the application of which appears to be promising in the fields of sensors [1], optics [2], anticounterfeiting technology [3–5], light-emitting diodes (LEDs) [6], and bioimaging [7–9]. Long-persistent luminescence is typically achieved using pure inorganic materials, such as the incorporation of transition metals and rare-earth elements into a host matrix, which has largely expanded the phosphor family. However, their relatively high cost, low flexibility, and complex methods (e.g., high synthetic temperature) [10, 11] have restricted their large-scale applications to some extent. The persistent luminescence of purely organic materials is generally less efficient due to their poor intersystem crossing (ISC) ability and their rapid rate of nonradiative deactivation caused by the molecular motions, including rotation, vibration, and collision. In recent years, an extensive collection of organic persistent phosphors with ultralong room-temperature phosphorescence (URTP) has been developed utilizing different design strategies, such as crystal engineering for tuning molecular stacking [12–15], H-aggregation [16, 17], formations of a metal-organic framework (MOF) [18, 19], carbon dots [20, 21], hybrid perovskites [22, 23], amorphous organic materials [24, 25], and many others [26–28].

Despite the continuous development of URTP systems, achieving a tunable and multicolor emission is still a challenge, particularly for those based on a single chromophore [29]. Doping of different contents of organic phosphors into polymers has been proven as an effective way to tune the RTP; however, the tunable emission range is still limited [30]. Furthermore, the RTP quantum yield is rather low compared with their inorganic counterparts, which has largely restricted the efficient visualization of long-persistent luminescence. Based on the advantageous characteristics of the inorganic solid-state framework, including its high rigidity and stability, together with the tuning variability of organic RTP molecules, we hypothesize that if the organic RTP phosphor guest can be doped into the inorganic host, the molecular motions and vibrations can be highly restricted, which would further suppress the nonradiative loss of triplet excitons and facilitate the RTP output [31]. Moreover, the RTP could be further tuned based on both host-guest interactions, variable molecular aggregation, and the external doping of inorganic ions. Until now, the organic-doped inorganic framework for a colorful ultralong RTP has only been speculated, which is likely due to the weak compatibility between organic/inorganic interfaces, leading to the great difficulty of organic doping.

**Figure 1:**
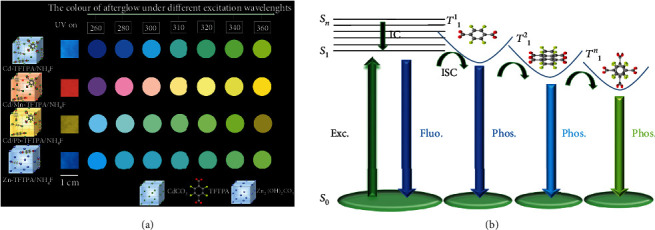
(a) Doped structures of Cd-TFTPA/NH_4_F, Cd/Mn-TFTPA/NH_4_F, Cd/Pb-TFTPA/NH_4_F, and Zn-TFTPA/NH_4_F samples as well as their corresponding long-persistent emission images under various excitation wavelengths. (b) The proposed mechanism of hybrid material for multicolor ultralong phosphorescence with the change of excitation wavelengths. The triplet excitons are generated from the singlet excitons through the intersystem crossing, enabling molecular phosphorescence owing to the strict suppression of the molecular motion by the inorganic matrix framework. The formation of different arrangements and stacking modes can induce different triplet excitons and lead to different phosphorescence.

In this work, tetrafluoroterephthalic acid (TFTPA) and CdCO_3_ were selected as the doped RTP guest and inorganic host, respectively, based on the following design factors: the terephthalic unit not only improves the intersystem crossing (ISC) rate to boost triplet excitons but also replaces part of the carbonate group in the inorganic matrix materials due to its strong acidity (pK_a_ = 1.17) and similar coordination ability. The strong electronegativity of the fluorine atoms in TFTPA can adjust the molecular orbital distributions and enhance the host-guest interactions, which favors the formation of multiple energy levels for a diverse colorful emission [32, 33]. Furthermore, using the heavy atom Cd as the metal unit in the inorganic matrix could be beneficial for mixing the singlet and triplet states of different electronic configurations and promoting energy transfer between different states, which favors the *n*–*π*∗ transition and enhances the RTP quantum yield. A series of codoped organic/inorganic hybrid materials were obtained using a facile hydrothermal method. Different arrangement modes of TFTPA and codoped metal ions formed in the inorganic materials establish different excited state energy levels for multicentral persistent luminescence. The hybrid materials present an excitation-dependent colorful long-lived RTP with tunable time-resolved lifetimes and quantum yields, which can be further used for information storage and multiple-channel encryption (Figure 1(a)). Therefore, this work provides a new platform to construct organic-doped hybrid materials with both a static and dynamic tunable URTP, towards multicolor long-persistent luminescence and information safety applications.

## 2. Results

**Scheme 1:**
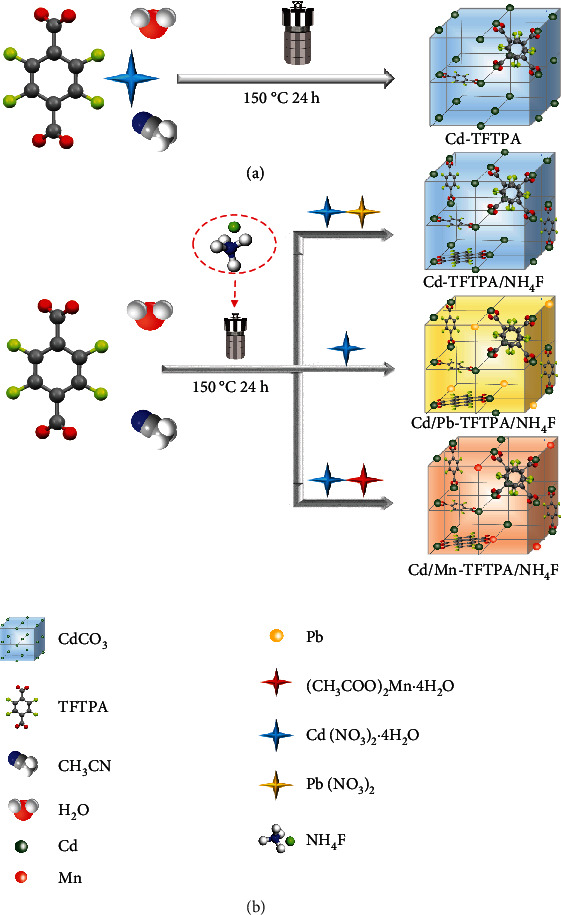
Synthetic routes to (a) Cd-TFTPA, (b) Cd-TFTPA/NH_4_F, Cd/Pb-TFTPA/NH_4_F, and Cd/Mn-TFTPA/NH_4_F.

The organic TFTPA doped CdCO_3_ matrix (Cd-TFTPA) is prepared via a one-pot hydrothermal process (Scheme 1(a)). The structure of the Cd-TFTPA is characterized by both single-crystal and powder X-ray diffraction (XRD), which show that the crystal pattern of the as-synthesized Cd-TFTPA is in good agreement with that of the standard CdCO_3_ (Figure S1). The typical scanning electron microscopy (SEM) images show a regularly polyhedral morphology with a size range of 10−15 *μ*m (Figure S2). The energy-dispersive elemental mapping demonstrates that the Cd, C, O, and F compositions are distributed homogeneously within the Cd-TFTPA (Figure S2), confirming that the TFTPA molecules are highly dispersed within the CdCO_3_ frameworks, with a content of 1.39% wt. As observed in the XPS survey spectra, the peaks centered at 412 and 405 eV are attributed to the Cd 3d_3/2_ and Cd 3d_5/2_ of the Cd^2+^ in Cd-TFTPA. F 1s for Cd-TFTPA is fitted with a peak located at 684.4 eV, which is assigned to the C−F [34]. The large peak in the C spectrum is dominated by two different structures of carbon: C_1_ located at 284.6 eV can be attributed to the C−C/C=C from the benzene of TFTPA; C_2_ centered at 289.3 eV is attributed to the O−C=O of TFTPA (Figure S3). The occurrence of the functional groups C=C, C−C, C−F, and O−C=O by the XPS test also indicates that the TFTPA molecules are highly confined within the Cd-TFTPA crystal lattice.

Upon excitation of the Cd-TFTPA with UV from 250 to 370 nm, the Cd-TFTPA fluorescence emission is mainly located at 445 to 455 nm (Figure S4). However, for the decay persistent emission (gating time 0.5 ms), with a change in the excitation wavelength from 270 to 370 nm, the long-lived RTP exhibits an obvious red-shift from sky-blue to yellow-green along with the main peak varying from 445 to 533 nm. The color variation of the Cd-TFTPA sample is further confirmed using the Commission Internationale de l'Eclairage (CIE) color coordinates under different excitation wavelengths. When the excitation wavelength changes from 260 to 360 nm, the color changes from a sky-blue (color coordination: (0.1736, 0.1656)) to yellow-green (color coordination: (0.3638, 0.5052)) with good linearity of the CIE coordinates (Figure S5f). The time-resolved phosphorescence spectra demonstrate that the RTP lifetimes are in the range of 107-290 ms (Figure S5c, d). To detect the origin of excitation-dependent RTP properties of the material, we performed the thermostimulated luminescence (TSL) test of the pure CdCO_3_ and Cd-TFTPA (Figure S6a). A lack of trap states was observed, which excludes the possibility that wavelength-dependent long-persistent properties of the material are derived from the inorganic matrix and support them being from the RTP of the doped TFTPA molecules (Figure S6b). Furthermore, a series of temperature-dependent steady-state and time-resolved spectrum measurements are systematically performed (Figure S7). With an increase in temperature, the emission at 440 nm and 533 nm gradually decreases in both intensity and excited lifetime, which is consistent with the trend of a decreased lifetime and spectral intensity for the conventional molecule-based RTP materials with increasing temperature [12]. In addition, the phosphorescent property of TFTPA is also investigated in a dilute solution of tetrahydrofuran (THF) at 77 K (Figure S8). The TFTPA molecule exhibits a broad blue emission band at around 452 nm when changing the excitation wavelength, and thus, the blue long-persistent luminescence of Cd-TFTPA can be ascribed to the RTP of the isolated molecules, as is observed in the solution. The yellow-green ultralong RTP is observed only in the hybrid materials due to the diversity of the guest molecular aggregation in the robust organic-inorganic hybrid materials. By virtue of the strong acidity and strong coordination ability of TFTPA, various arrangements and stacking modes of the TFTPA molecules appear within the CdCO_3_ crystal lattices, which lead to different triplet energy levels, and thus endow the hybrid materials with an excitation-dependent colorful RTP.

To further tune the doped contents of the TFTPA, the Cd-TFTPA has been modified through the addition of NH_4_F during the hydrothermal synthesis (Scheme 1(b)), since NH_4_F can serve as an etchant or surface modifier to expose more metal binding sites and structural defects during the synthesis process [35, 36]. The structure of the as-obtained sample of Cd-TFTPA/NH_4_F is confirmed by the powder XRD pattern, which exhibits that the addition of NH_4_F does not induce a structure change relative to the Cd-TFTPA (Figure S9). The ^19^F NMR spectra show that compared with the pristine TFTPA with a chemical shift at 140 ppm, the case for Cd-TFTPA/NH_4_F shifts to a high field at 168 ppm. This behavior can be related to the removal of hydrogen in TFTPA and the strong coordination with Cd ions (Figure S10). SEM reveals that the Cd-TFTPA/NH_4_F maintains a similar size (10−15 *μ*m) relative to the Cd-TFTPA, while its surface is obviously more defective than the Cd-TFTPA (Figure S11), suggesting that the NH_4_F has a tremendous etching effect on the micro-/nanostructure of the Cd-TFTPA/NH_4_F. Figure S11 shows the representative EDX elemental mapping of the Cd-TFTPA/NH_4_F, indicating the coexistence and uniform distribution of Cd, C, O, and F throughout the sample (Figure S11), and the doped content of TFTPA in the Cd-TFTPA/NH_4_F sample (3.36% wt) is higher than that in the Cd-TFTPA, suggesting that the addition of NH_4_F does create more defects and introduces more TFTPA molecules. XPS is further used to study the surface states and chemical composition of the Cd-TFTPA/NH_4_F. The elements Cd, C, O, and F contained in the Cd-TFTPA sample are also present in Cd-TFTPA/NH_4_F (Figure S12). The peak area of the F element from Cd-TFTPA/NH_4_F is larger than the Cd-TFTPA sample, which is consistent with more TFTPA molecules being doped into the CdCO_3_ crystal.

**Figure 2:**
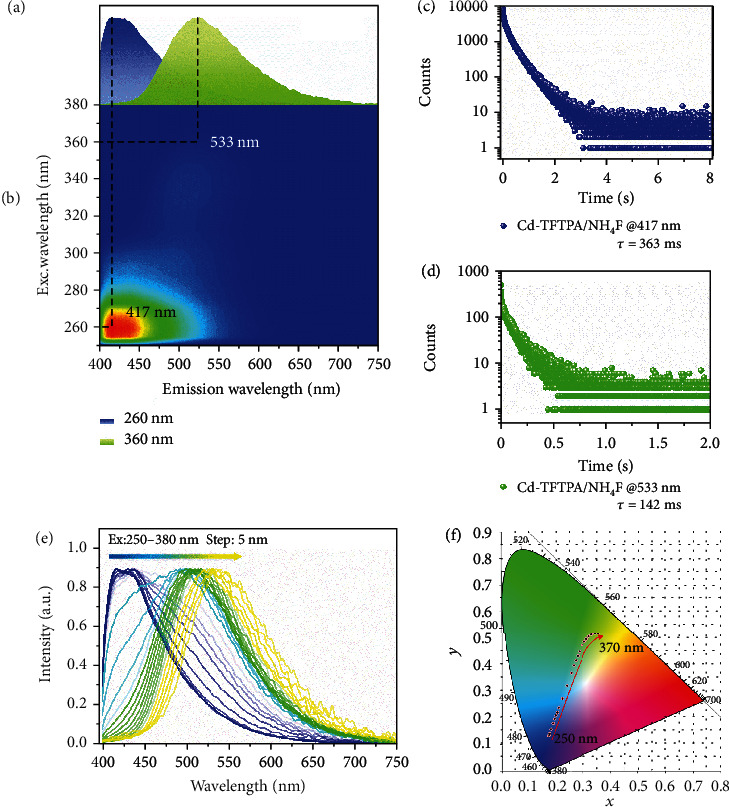
Photoluminescence characterization of Cd-TFTPA/NH_4_F powder under ambient conditions. (a) The URTP spectra of the Cd-TFTPA/NH_4_F powder under the excitation at 260 nm (blue) and 360 nm (green), respectively. (b) Excitation-phosphorescence mapping of powder under ambient conditions. (c, d) Decay curves of Cd-TFTPA/NH_4_F at 417 nm and 533 nm. (e) Excitation-dependent phosphorescence spectra of Cd-TFTPA/NH_4_F. (f) CIE coordinate diagram of Cd-TFTPA/NH_4_F by changing the excitation wavelengths.

To detect how different contents of doped TFTPA in the host framework influence the ultralong RTP, the photophysical properties of Cd-TFTPA/NH_4_F have been investigated under ambient conditions. When excited by UV light at 250 nm, Cd-TFTPA/NH_4_F shows a blue-white emission in the solid state, with a major emission peak at 420 nm (Figure S13a). After the removal of the excitation source, a dark blue long-lived photoemission is observed (*λ*_max_ = 417 nm) with an RTP quantum yield of 26.1% (Figure S14) and an ultralong lifetime of 360 ms (Figure 2(c)). Particularly, its RTP lifetime is 313 times longer than that of the pristine TFTPA (Figure S15), suggesting that the use of doping into an inorganic framework can be an efficient way to prolong the excitation lifetime of molecular phosphors. Upon excitation at 360 nm, it shows similar fluorescence located at 420 nm, but a different yellow-green ultralong RTP (*λ*_max_ = 533 nm) with a lifetime of 142 ms (Figure 2(d)). With increasing the excitation wavelength from 250 nm to 370 nm, the Cd-TFTPA/NH_4_F has a wide emission wavelength range (from 417 nm to 545 nm) with corresponding CIE coordinate changes from (0.1713, 0.1337) to (0.3557, 0.5148) (Figure 2(f)) and tunable RTP lifetimes (363 ms and 134 ms for 417 nm and 545 nm). TSL text for the Cd-TFTPA/NH_4_F samples similarly proves that the wavelength-dependent properties of the material are not derived from the inorganic host matrix (Figure S6c).

To further understand the origin of the RTP emission, a comparison of the UV-vis absorption spectra of the hybrid materials has been carried out (Figure S16). Compared to the pristine CdCO_3_, the Cd-TFTPA and Cd-TFTPA/NH_4_F show two peaks around 255 nm and 285 nm, which can be attributed to the *π*‐*π*∗ transitions in the benzene core; a low-intensity absorption peak around 350 nm is ascribed to the *n*‐*π*∗ transition of the functional groups of C=O. The overlap of the RTP excitation and absorption spectrum at 300–400 nm indicates that the RTP primarily originates from the C=O bond of TFTPA. Therefore, compared with Cd-TFTPA, Cd-TFTPA/NH_4_F shows a longer RTP lifetime and a wider excitation-dependent spectral range, which can be attributed to more TFTPA molecules being doped into the inorganic matrix. With the formation of a richer arrangement and stacking mode, more excited state levels were generated.

To obtain a deeper understanding of the role of doping of the RTP molecule TFTPA within the inorganic matrix framework, we have further prepared the samples with the TFTPA absorbed at the CdCO_3_ surface through the hydrothermal reactions using pure CdCO_3_, TFTPA, and NH_4_F as reagents, with the same reaction conditions as those of Cd-TFTPA (named TFTPA@CdCO_3_ and TFTPA@CdCO_3_/NH_4_F). Their RTP performances have been measured under different excitation wavelengths (Figure S17a, c, e). The delayed PL spectra of the pristine CdCO_3_ reveal that the phosphorescence signal (452 nm) excited by 280-360 nm has a very short lifetime of 4.38 *μ*s under ambient conditions (Figure S17b). Moreover, TFTPA@CdCO_3_ and TFTPA@CdCO_3_/NH_4_F exhibit largely reduced RTP lifetimes of 1 ms and 10.43 ms, respectively, which are reduced by 99.65% and 97.13% compared with the Cd-TFTPA and Cd-TFTPA/NH_4_F (Figure S17d, f), confirming the important role in the doping of organics into a host matrix. We suggest that, in both TFTPA@CdCO_3_ and TFTPA@CdCO_3_/NH_4_F, only a small amount of TFTPA molecules is adsorbed at the surface of CdCO_3_, while for the hybrid materials (Cd-TFTPA and Cd-TFTPA/NH_4_F), the formation of high confinement and rigidity conditions for TFTPA within the CdCO_3_ crystal lattice during the crystal growth would result in a much longer RTP lifetime and a wider wavelength range. Owing to the higher tunability of RTP performance, we chose the Cd-TFTPA/NH_4_F as the key material for a more detailed study in the following section.

Stability is highly important for organic-inorganic hybrid materials in practical RTP applications. To study the luminescent stability of Cd-TFTPA/NH_4_F under solution conditions, we firstly detected the persistent RTP underwater. It is observed that the long-persistent luminescence can be maintained underwater (pH = 7). The RTP decay lifetime values of Cd-TFTPA/NH_4_F underwater are determined to be 345 and 132 ms at the wavelengths of 254 nm and 365 nm, respectively (Figure S18b, d), and the blue light and yellow-green afterglow of Cd-TFTPA/NH_4_F can be easily observed (inset in Figure S18a, c). In addition, the sample exhibits the same RTP features under both oxygen and nitrogen atmospheres (Figure S18e). We also detected luminescent stability of the hybrid materials under extreme conditions. Although the RTP intensity of the materials changes in acidic or basic environments (pH = 1-13), its RTP lifetime does not decline substantially, as shown in Figure S19. Based on the PXRD patterns (Figure S20), the Cd-TFTPA/NH_4_F at pH = 1-13 could largely retain their structures after being socked with hydrochloric acid and sodium hydroxide solutions. In our opinion, the organic RTP units are effectively protected by the CdCO_3_ inorganic matrix, and the strong coordination ability of the organic TFTPA with Cd^2+^ in the hybrid materials improves the RTP resistance under oxygen and acidic/alkaline conditions.

It is reported that the doping of different active metal ions can tune the luminescent properties of host materials to a large extent by changing the electronic structure [37]. Doping or codoping with metal ions can modify and/or enrich the lattice structure, leading to interesting and highly diverse photonic performances [38–40]. Based on the expectation that effective energy transfer between the organic TFTPA and the dopant ions results in strong sensitized metal ions, we further introduced typical photoemission central (Mn^2+^ and Pb^2+^ ions with 0.7% and 3.4% molar content) using an *in situ* codoping method during the formation of Cd-TFTPA/NH_4_F. The PXRD patterns of the as-synthesized Cd/Mn-TFTPA/NH_4_F and Cd/Pb-TFTPA/NH_4_F are in good agreement with the simulated results from that of Cd-TFTPA, confirming their isostructures (Figure S21). Thus, the doping of a small amount of Mn^2+^ or Pb^2+^ does not cause structural changes in the materials. For the Cd/Mn-TFTPA/NH_4_F and Cd/Pb-TFTPA/NH_4_F, SEM images show a similarity to the Cd-TFTPA/NH_4_F sample in both sample size and surface defective microstructures (Figures S22 and S23). EDX element mapping shows that the TFTPA molecules, Mn^2+^, and Pb^2+^ ions are uniformly doped into the materials (Figures S22 and S23). The XPS test proves that Mn^2+^ and Pb^2+^ are successfully doped in the samples (Figures S24 and S25).

**Figure 3:**
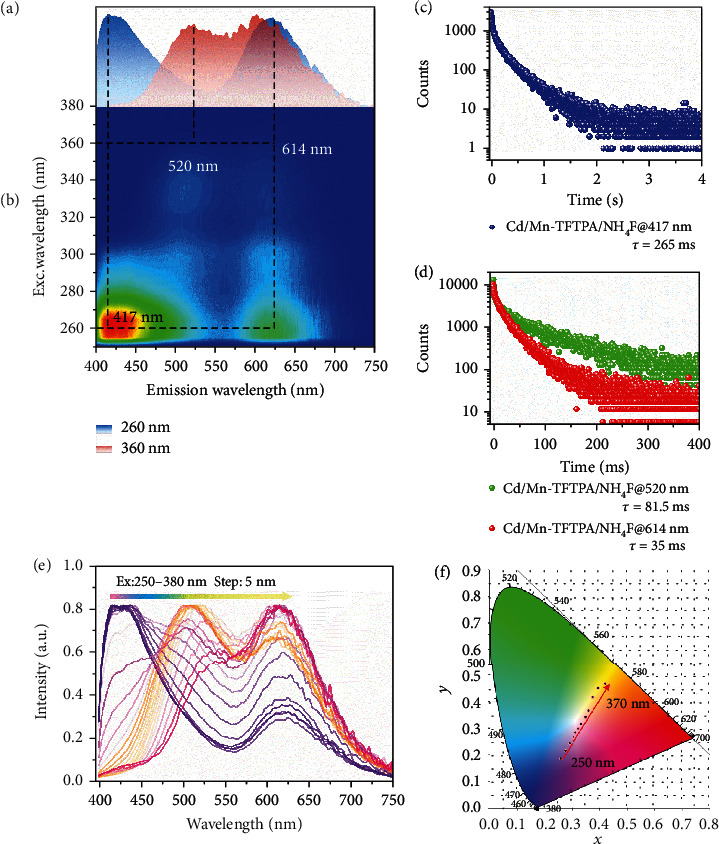
Photoluminescence characterization of Cd/Mn-TFTPA/NH_4_F powder under ambient conditions. (a) The URTP spectra of the Cd/Mn-TFTPA/NH_4_F powder under the excitation at 260 nm (blue) and 360 nm (red), respectively. (b) Excitation-phosphorescence mapping of powder under ambient conditions. (c, d) Decay curves of Cd/Mn-TFTPA/NH_4_F at 417 nm, 520 nm, and 614 nm. (e) Excitation-dependent phosphorescence spectra of Cd/Mn-TFTPA/NH_4_F. (f) CIE coordinate diagram of Cd/Mn-TFTPA/NH_4_F by changing the excitation wavelengths.

**Figure 4:**
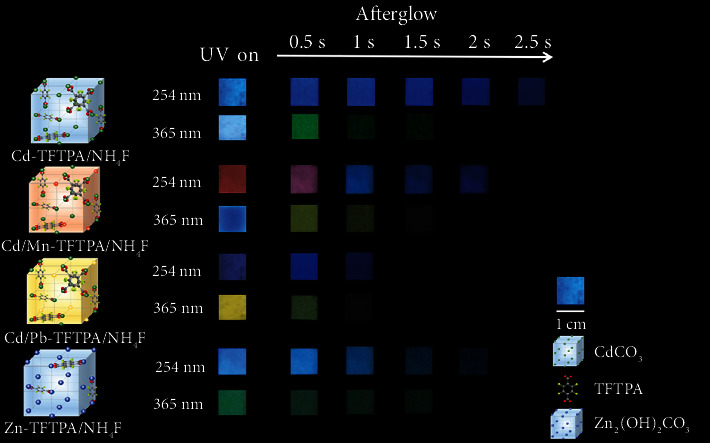
Photographs of the RTP materials taken before and after turning off of the different excitation wavelengths (254 nm and 365 nm) in the scale from 0 to 2.5 s.

Due to the effective doping of Mn^2+^ within Cd-TFTPA/NH_4_F, upon UV excitation at 250 nm, the persistent luminescence of the Cd/Mn-TFTPA/NH_4_F exhibits three peaks at 417, 520, and 614 nm (Figures 3(a) and 3(b)). Compared with the Cd-TFTPA/NH_4_F, Cd/Mn-TFTPA/NH_4_F exhibits an additional red emission peak at 614 nm, which can be assigned to the characteristic emission of Mn^2+^ ions due to the ^4^T_1_-^6^A_1_ transition. Increasing the excitation wavelength from 250 to 370 nm results in dynamic changes in the three peaks, with the relative intensity in both blue and green ranges decreasing gradually, while the intensity of the red region increases systematically (Figure S26b), suggesting that more excitation energy at a longer wavelength sensitizes the Mn^2+^ to achieve an effective energy transfer (Figure S29). The corresponding CIE coordinate shifts from (0.2361, 0.559) to (0.4349, 0.4645) (Figure 3(f)). Based on the time-resolved decay spectra, the lifetime values of Cd/Mn-TFTPA/NH_4_F are 35 ms and 265 ms at 614 nm and 417 nm, respectively. The obvious differences in the persistent luminescence indicate that the double RTP can be tuned dynamically, in which the pink afterglow changes into a blue color after 0.5 seconds upon turning off the UV at 254 nm, which can be easily recognized by the naked eye. To the best of our knowledge, such systems, with a tunable RTP color in the time-removed scale, are still limited to date [41]. Thus, our results can be further applied as a new type of optical information storage and multidimensional anticounterfeiting materials (Figure 4).

For the Cd/Pb-TFTPA/NH_4_F, upon excitation at 254 nm, the RTP of both the blue and green emission is fairly balanced, which decreases simultaneously as the excitation wavelength increases (Figure S28e). The yellow wavelength position begins to appear with a UV excitation at 350 nm, which is due to more effective sensitizing of the luminescence of Pb^2+^ at the long-wavelength excitation energy (Figure S30). The corresponding CIE coordinate value varies from (0.2552, 0.2823) to (0.3893, 0.522) (Figure S28f). Therefore, the obtained organic-inorganic hybrid materials (Cd/Mn-TFTPA/NH_4_F and Cd/Pb-TFTPA/NH_4_F) present a dynamic tunable character for multicentral RTP emission (Figures S31 and S32), and the excitation-dependent long-persistent luminescence of an organic-doped host matrix can be extended to other ion codoped systems achieving a high-efficiency multicolor RTP.

To confirm the versatility of our methodology for the organic-doped inorganic frameworks, Cd(NO_3_)_2_·4H_2_O is replaced with Zn(NO_3_)_2_·6H_2_O, and Zn-based hybrid materials can be obtained (separately named Zn-TFTPA and Zn-TFTPA/NH_4_F) using the same hydrothermal method due to the same subfamily of the Zn^2+^ and Cd^2+^ (Scheme S1a, b). The PXRD patterns of Zn-TFTPA and Zn-TFTPA/NH_4_F are in agreement with that of Zn_2_(OH)_2_CO_3_ (the JCPDS #11-0287) (Figure S33). A series of characterization methods, such as XPS, SEM, and EDX elemental mapping, confirm the successful doping of TFTPA into the Zn-based host matrix (Figures S34 and S35). Both Zn-TFTPA and Zn-TFTPA/NH_4_F samples show a tunable ultralong RTP from cyan to yellow-green by varying the excitation wavelength from 250 to 370 nm (Figure 4, Figure S38 b, d). For Zn-TFTPA, the luminescence is centered at 460 nm with a lifetime of 6.6 ms (Figure S39). For Zn-TFTPA/NH4F, the luminescence peak is located at 465 nm with a lifetime of 261 ms (Figure S40) with a maximum RTP efficiency of the Zn-TFTPA/NH_4_F of 43.9% (Figure S14), which is much higher than that of the pristine TFTPA sample, and also among the high level for the state-of-the-art RTP materials [42, 43]. The two Zn-based hybrids represent a certain universality of this uniform organic doping method via a hydrothermal process, which provides a new strategy to obtain tunable RTP materials with excitation wavelength dependence.

Data security is of great significance to human society. Recently, benefitting from their long-lived luminescence, RTP materials have received more and more attention from their broad prospects for security protection such as information storage, encryption, and anticounterfeiting. However, a single RTP color for typical materials has made it difficult to design multiple levels of protection for confidential information. Based on the excitation-dependent and dynamic tunable RTP of the organic-inorganic hybrids in this work, we further demonstrate its potential applications in multiple anticounterfeiting and information encryption. In contrast to the previous information encryption mode based on RTP properties, herein, we provide a more efficient and time-resolved multi-information encryption program.

**Figure 5:**
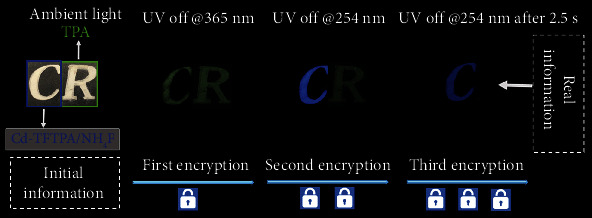
Schematic diagram depicting the evolution from triple information encryption to decryption.

As shown in Figure 5, the encryption model of word “CR” is designed, in which “C” includes the hybrid material Cd-TFTPA/NH_4_F and “R” consisted of TPA (terephthalic acid) only with a single yellow-green RTP. The TPA and Cd-TFTPA/NH_4_F could exhibit different afterglow colors upon different UV excitation. The encryption process is outlined as follows: in the first round of encryption, after turning off the UV lamp at 365 nm, only the “CR” shows a yellow-green afterglow; in the second round of encryption, when the 254 nm UV lamp is turned off, “C” shows a blue afterglow; in the third round of encryption, after 2.5 seconds upon turning off the UV lamp at 254 nm, the blue afterglow still exists, indicating that “C” is the real information that is ultimately needed. The letter “C” stands for the final true message through three continuous encryption processes. This extra layer of information enhances the overall level of security and yields a triple encryption system. Thus, the excitation-dependent RTP and time-resolved color change of hybrid materials are good candidates for multiple information security applications.

**Figure 6:**
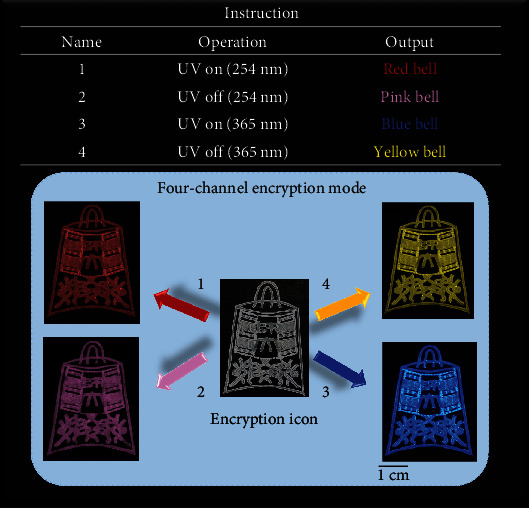
The application of Cd/Mn-TFTPA/NH_4_F in security protection.

Furthermore, based on the obvious differences in the luminescent colors before and after ceasing the UV excitation at 254 nm and 365 nm, we have further developed a kind of multichannel encryption technology for optical anticounterfeiting applications, which exhibit a high level of security as compared to the typical single encryption mode. A bell icon can be facilely manufactured using a simple screen-printing process with Cd/Mn-TFTPA/NH_4_F powdered materials. Considering the emitting colors can be clearly distinguished with varied excitation wavelengths, four sorts of luminescence, which represent the output information (red, pink, blue, and yellow, Figure 6), can be readily observed by the naked eye. Under the confidential guidance, upon treating the encryption bell with four operations in the precise order (UV on (254 nm and 365 nm) and UV off (254 and 365 nm)), only when the corresponding red, pink, blue, and yellow colors can be identified will the encryption process be regarded as a success. Although this example only has one operating order, a similar process is also suitable for other materials, such as Cd/Pb-TFTPA/NH_4_F, confirming the high degree of flexibility in design for the excitation-dependent colorful RTP materials for use in information anticounterfeiting.

## 3. Conclusion

In summary, a universal strategy through organic guest-doped inorganic frameworks is developed to obtain new types of hybrid materials featuring a wide-range, color-tunable, and long-persistent luminescence. The obtained hybrids present obviously increased RTP lifetimes, high quantum yields, and enhanced luminescence stability as compared to the pristine organic phosphor. With a change in the excitation, the emission color of these hybrid materials can be tuned from dark blue to yellow-green under ambient conditions, with a wide range of ultralong RTP tunability greater than 120 nm, which may set a record among the state-of-the-art tunable RTP ranges with excitation dependence. Furthermore, the excitation-dependent RTP properties can also be extended to other metal ion codoped systems towards varied multicolor RTP and dynamical RTP changes in the time-resolved scale, which endow great opportunities for multidimensional data encryption and information anticounterfeiting applications. Thus, compared with the well-developed purely organic RTP systems, the molecular hybrids integrated with both organics and inorganics serve as a highly tunable way to adjust the RTP performances. It can be expected that by virtue of the facile organic doping method via the simple hydrothermal process, both the static and dynamic tunable RTP can be further extended to other systems for the development of colorful long-persistent luminescence.

## Data Availability

The data used to support the findings of this study are included within the article and the supplementary materials.

## Supplementary Materials

Supplementary Materials Scheme S1: synthetic routes to Zn-TFTPA and Zn-TFTPA/NH_4_F. Figure S1: PXRD patterns for Cd-TFTPA. Figure S2: SEM and EDX mapping for corresponding elemental distributions in Cd-TFTPA. Figure S3: XPS of Cd-TFTPA. Figure S4: fluorescence spectra of Cd-TFTPA. Figure S5: photoluminescence characterization of Cd-TFTPA powder under ambient conditions. Figure S6: TSL text of pure CdCO_3_, Cd-4FTPA, and Cd-TFTPA/NH_4_F. Figure S7: the temperature-dependent delayed PL spectra and time-resolved PL-decay profiles of Cd-TFTPA under different temperatures at 440 nm and 533 nm. Figure S8: phosphorescence spectra of TFTPA in a dilute solution of THF under 280, 320, and 350 nm excitation at 77 K. Figure S9: the PXRD patterns for Cd-TFTPA/NH_4_F. Figure S10: ^19^F NMR of TFTPA and Cd-TFTPA/NH_4_F. Figure S11: SEM and EDX mapping for Cd-TFTPA/NH_4_F. Figure S12: XPS of Cd-TFTPA/NH_4_F. Figure S13: fluorescence spectra and delayed PL spectra of Cd-TFTPA/NH_4_F. Figure S14: the comparison of PLQY values for Cd-TFTPA, Zn-TFTPA, Cd-TFTPA/NH_4_F, and Zn-TFTPA/NH_4_F under different wavelengths. Figure S15: fluorescence spectra, delayed PL spectra, and decay curves of TFTPA at 456 nm. Figure S16: solid-state UV-vis absorption spectra of CdCO_3_, TFTPA, Cd-TFTPA, Cd-TFTPA/NH_4_F, and Zn-TFTPA/NH_4_F under ambient conditions. Figure S17: the delayed PL spectra of CdCO_3_, CdCO_3_@TFTPA, and CdCO_3_@TFTPA/NH_4_F; the delayed lifetime decay profiles at 452 nm of CdCO_3_, 440 nm of CdCO_3_@TFTPA, and 427 nm of CdCO_3_@TFTPA/NH_4_F. Figure S18: delayed PL spectra of Cd-TFTPA/NH_4_F detected in wet and dry conditions, the delayed lifetime decay profiles of Cd-TFTPA/NH_4_F at 417 nm and 533 nm, different atmospheric environments, and the delayed lifetime decay profiles of Cd-TFTPA/NH_4_F at 417 nm. Figure S19: delayed PL spectra of Cd-TFTPA/NH_4_F detected under different pH, the delayed lifetime decay profiles of Cd-TFTPA/NH_4_F at 417 nm and 525 nm under different pH. Figure S20: PXRD patterns of Cd-TFTPA/NH_4_F under different pH. Figure S21: PXRD patterns for Cd-TFTPA/NH_4_F, Cd/Mn-TFTPA/NH_4_F, and Cd/Pb-TFTPA/NH_4_F. Figure S22: SEM and EDX mapping of Cd/Mn-TFTPA/NH_4_F. Figure S23: SEM and EDX mapping of Cd/Pb-TFTPA/NH_4_F. Figure S24: XPS of Cd/Mn-TFTPA/NH_4_F. Figure S25: XPS of Cd/Pb-TFTPA/NH_4_F. Figure S26: fluorescence spectra and delayed PL spectra of Cd/Mn-TFTPA/NH_4_F. Figure S27: fluorescence spectra of Cd/Pb-TFTPA/NH_4_F. Figure S28: schematic diagram for the energy levels of Cd/Mn-TFTPA/NH_4_F. Figure S29: schematic diagram for the energy levels of Cd/Pb-TFTPA/NH_4_F. Figure S30: photoluminescence characterization of Cd/Pb-TFTPA/NH_4_F. Figure S31: fluorescent and phosphorescence CIE diagram of sample Cd/Mn-TFTPA/NH_4_F under different excitation wavelengths. Figure S32: fluorescent and phosphorescence CIE diagram of sample Cd/Pb-TFTPA/NH_4_F under different excitation wavelengths. Figure S33: the JCPDS (#11-0287) and as-synthesized PXRD patterns for Zn-TFTPA and Zn-TFTPA/NH_4_F. Figure S34: XPS of Zn-TFTPA. Figure S35: XPS of Zn-TFTPA/NH_4_F. Figure S36: SEM and EDX mapping for Zn-TFTPA. Figure S37: SEM and EDX mapping for Zn-TFTPA/NH_4_F. Figure S38: photoluminescence characterization of Zn-TFTPA and Zn-TFTPA/NH_4_F powder. Figure S39: fluorescence spectra, delayed PL spectra, and decay curves of Zn-TFTPA at 460 nm and 522 nm. Figure S40: fluorescence spectra, delayed PL spectra, and decay curves of Zn-TFTPA/NH_4_F at 465 nm and 530 nm. Table S1: phosphorescence lifetimes (*τ*) of Cd-TFTPA, Cd-TFTPA/NH_4_F, Cd/Mn-TFTPA/NH_4_F, Cd/Pb-TFTPA/NH_4_F, Zn-TFTPA, and Zn-TFTPA/NH_4_F.
