# Supplementary material for: Room-Temperature Phosphorescent Organic-Doped Inorganic Frameworks Showing Wide-Range and Multicolor Long-Persistent Luminescence
Source: Research (Wash D C). 2021 Apr 9;2021:9862327. doi: 10.34133/2021/9862327 (PMC8053305; doi:10.34133/2021/9862327)
Supplement: Supplementary Materials — Scheme S1: synthetic routes to Zn-TFTPA and Zn-TFTPA/NH4F. Figure S1: PXRD patterns for Cd-TFTPA. Figure S2: SEM and EDX mapping for corresponding elemental distributions in Cd-TFTPA. Figure S3: XPS of Cd-TFTPA. Figure S4: fluorescence spectra of Cd-TFTPA. Figure S5: photoluminescence characterization of Cd-TFTPA powder under ambient conditions. Figure S6: TSL text of pure CdCO3, Cd-4FTPA, and Cd-TFTPA/NH4F. Figure S7: the temperature-dependent delayed PL spectra and time-resolved PL-decay profiles of Cd-TFTPA under different temperatures at 440 nm and 533 nm. Figure S8: phosphorescence spectra of TFTPA in a dilute solution of THF under 280, 320, and 350 nm excitation at 77 K. Figure S9: the PXRD patterns for Cd-TFTPA/NH4F. Figure S10: 19F NMR of TFTPA and Cd-TFTPA/NH4F. Figure S11: SEM and EDX mapping for Cd-TFTPA/NH4F. Figure S12: XPS of Cd-TFTPA/NH4F. Figure S13: fluorescence spectra and delayed PL spectra of Cd-TFTPA/NH4F. Figure S14: the comparison of PLQY values for Cd-TFTPA, Zn-TFTPA, Cd-TFTPA/NH4F, and Zn-TFTPA/NH4F under different wavelengths. Figure S15: fluorescence spectra, delayed PL spectra, and decay curves of TFTPA at 456 nm. Figure S16: solid-state UV-vis absorption spectra of CdCO3, TFTPA, Cd-TFTPA, Cd-TFTPA/NH4F, and Zn-TFTPA/NH4F under ambient conditions. Figure S17: the delayed PL spectra of CdCO3, CdCO3@TFTPA, and CdCO3@TFTPA/NH4F; the delayed lifetime decay profiles at 452 nm of CdCO3, 440 nm of CdCO3@TFTPA, and 427 nm of CdCO3@TFTPA/NH4F. Figure S18: delayed PL spectra of Cd-TFTPA/NH4F detected in wet and dry conditions, the delayed lifetime decay profiles of Cd-TFTPA/NH4F at 417 nm and 533 nm, different atmospheric environments, and the delayed lifetime decay profiles of Cd-TFTPA/NH4F at 417 nm. Figure S19: delayed PL spectra of Cd-TFTPA/NH4F detected under different pH, the delayed lifetime decay profiles of Cd-TFTPA/NH4F at 417 nm and 525 nm under different pH. Figure S20: PXRD patterns of Cd-TFTPA/NH4F under different pH. Figure S21: PXRD [file 9862327.f1.docx]

**Room-temperature phosphorescent organics doped inorganic frameworks showing wide-range and multi-colorful long-persistent luminescence**

Guowei Xiao^1^, Bo Zhou^1^, Xiaoyu Fang^1^, and Dongpeng Yan^123^*

^1^Beijing Key Laboratory of Energy Conversion and Storage Materials, College of Chemistry Beijing Normal University, Beijing 100875, P. R. China.

^2^ College of Chemistry, Key Laboratory of Radiopharmaceuticals, Ministry of Education, Beijing Normal University, Beijing 100875, P. R. China

^3^College of Chemistry and Molecular Engineering, Zhengzhou University, Zhengzhou 450001, China

**Supporting Information**

**Table of Content**

1. **Experimental Procedures**
2. **Figures (Figure S1-Figure S40), Scheme S1 and Table S1**

**Materials and Reagents.** Analytically tetrafluoroterephthalic acid, pure Cd(NO_3_)_2_·4H_2_O, Zn(NO_3_)_2_·6H_2_O, Mn(CH_3_COO)_2_·4H_2_O, Pb(NO_3_)_2_, CdCO_3_, acetonitrile, ammonium fluoride and tetrahydrofuran were purchased from Sigma Chemistry Co. Ltd. and used without further purification.

**Synthesis of Cd-TFTPA and Cd-TFTPA/NH_4_F:** A mixture of Cd(NO_3_)_2_·4H_2_O (1 mmol, 0.236 g), TFTPA (0.5 mmol, 0.119 g), CH_3_CN (2 mL), and water (8 mL) was sealed in a 23 mL Teflon reactor, kept under autogenous pressure at 150 °C for 24 h, and then cooled with a speed of 10 °C min^−1^ to room temperature. Light yellow bulk crystals were filtered off, washed with distilled water and ethanol in turn, and dried in air, Yield: 50% (based on Cd). Cd-TFTPA/NH_4_F was synthesized using a similar method, but with adding 0.5mmol NH_4_F in the process of synthesis.

**Synthesis of Cd/Mn-TFTPA/NH_4_F:** A mixture of Cd(NO_3_)_2_·4H_2_O (1 mmol, 0.236 g), (CH_3_COO)_2_Mn·4H_2_O, (0.01 mmol, 0.0025g), TFTPA (0.5 mmol, 0.119 g), NH_4_F (0.5 mmol, 0.019g), CH_3_CN (2 mL), and water (8 mL) was sealed in a 23 mL Teflon reactor, kept under autogenous pressure at 150 °C for 24 h, and then cooled with a speed of 10 °C min^−1^ to room temperature. Yield: 45% (based on Cd).

**Synthesis of Cd/Pb-TFTPA/NH_4_F:** A mixture of Cd(NO_3_)_2_·4H_2_O (1 mmol, 0.236 g), Pb(NO_3_)_2_, (0.01mmol, 0.0033g), TFTPA (0.5 mmol, 0.119 g), NH_4_F (0.5 mmol, 0.019g), CH_3_CN (2 mL), and water (8 mL) was sealed in a 23 mL Teflon reactor, kept under autogenous pressure at 150 °C for 24 h, and then cooled with a speed of 10 °C min^−1^ to room temperature. Yield: 48% (based on Cd).

**Synthesis of Zn-TFTPA and Zn-TFTPA/NH_4_F:** Zn-TFTPA and Zn-TFTPA/NH_4_F were synthesized using a similar method with Cd-TFTPA and Cd-TFTPA/NH_4_F, but with Zn(NO_3_)_2_·6H_2_O instead of Cd(NO_3_)_2_·4H_2_O.

**Characterization**. Single-crystal X-ray diffraction data of these samples were collected at room temperature on Bruker SMART APEX CCD diffractometer employing monochromatized Mo Kα radiation (λ = 0.71073 Å). PXRD patterns of these samples were performed on a Rigaku Ultima-IV automated diffraction system with Cu Kα radiation (λ = 1.5406 Å), and the measurements were made in a 2*θ* range of 5°–70° at room temperature with a step of 0.02° (2*θ*) as well as scan speed of 5° min^−1^. The operating power was 40 kV, 30 mA. UV–vis absorption spectra were obtained on a Shimadzu UV-3600 spectrophotometer at room temperature. Data were collected in the wavelength range of 200–800 nm. BaSO_4_ powder was used as a standard sample (100% reflectance). Photographs for the six hybrid materials were taken under OLYMPUS IXTI fluorescence microscope. ^19^F-NMR spectras were recorded at 600 MHz on a JEOL-600 spectrometers at ambient temperature. All the relevant PL tests and time-resolved lifetime were conducted on an Edinburgh FLS980 fluorescence spec-trometer. The PLQY of the hybrid materials and tetrafluoroterephthalic acid (TFTPA) were reckoned by using a Tef-lon-lined integrating sphere (F-M101, Edinburgh, diameter: 150 mm and weight: 2 kg) accessory in FLS980 fluorescence spectrometer. Scanning electron microscopy with an accelerating voltage of 20 kV (SEM, Zeiss SUPRA 55) was used for detailed morphology analyses. X-ray photoelectron spectrometry (XPS) spectra were recorded using Al Kα radiation (Thermo VG ESCALAB MK II). Thermo-stimulated luminescence (TSL) was performed on 3D-TOSL (aidi ruisheng).

**Experimental Section**

**Scheme S1.** Synthetic routes to Zn-TFTPA (a), Zn-TFTPA/NH_4_F (b).

**Figure S1.** PXRD patterns for Cd-TFTPA.

**Figure S2.** Scanning electron microscopy (SEM) and Energy-dispersive X-ray spectrometry (EDX) mapping for corresponding elemental distributions in Cd-TFTPA.

**Figure S3.** X-ray photoelectron spectroscopy (XPS) of Cd-TFTPA.

**Figure S4.** Fluorescence spectra of Cd-TFTPA.

**Figure S5.** Photoluminescence characterization of Cd-TFTPA powder under ambient conditions.

**Figure S6.** The thermo stimulated luminescence (TSL) text of pure CdCO_3_ (a), Cd-4FTPA (b) and Cd-TFTPA/NH_4_F (c).

**Figure S7.** The temperature-dependent delayed PL spectra (a,c) and time-resolved PL-decay profiles of Cd-TFTPA under different temperature at 440 nm and 533 nm.

**Figure S8.** Phosphorescence spectra of TFTPA in a dilute solution of Tetrahydrofuran (THF) under 280, 320 and 350 nm excitation at 77 K.

**Figure S9.** The simulated (black) and as-synthesized (red) PXRD patterns for Cd-TFTPA/NH_~~4~~_F.

**Figure S10.** ^19^FNMR of TFTPA (a) and Cd-TFTPA/NH_4_F (b).

**Figure S11.** Scanning electron microscopy (SEM) and Energy-dispersive X-ray spectrometry (EDX) mapping for corresponding elemental distributions in Cd-TFTPA/NH_4_F.

**Figure S12.** X-ray photoelectron spectroscopy (XPS) of Cd-TFTPA/NH_4_F.

**Figure S13.** Fluorescence spectra (a) and delayed PL spectra (b) of Cd-TFTPA/NH_4_F.

**Figure S14.** The comparison of PLQY values for Cd-TFTPA, Zn-TFTPA, Cd-TFTPA/NH_4_F, Zn-TFTPA/NH_4_F under different wavelengths.

**Figure S15.** Fluorescence spectra (a), delayed PL spectra (b) and decay curves (c) of TFTPA at 456 nm.

**Figure S16.** Solid-state UV-vis absorption spectra of CdCO_3_, TFTPA and Cd-TFTPA, Cd-TFTPA/NH_4_F, Zn-TFTPA/NH_4_F under ambient conditions.

**Figure S17.** The delayed PL spectra of CdCO_3_ (a) CdCO_3_@TFTPA (b) CdCO_3_@TFTPA/NH_4_F (c), the delayed lifetime decay profiles at 452 nm of CdCO_3_ (b), 440 nm of CdCO_3_@TFTPA (d) and 427 nm of CdCO_3_@TFTPA/NH_4_F (f).

**Figure S18.** Delayed PL spectra of Cd-TFTPA/NH_4_F detected in wet and dry conditions (a, c), the delayed lifetime decay profiles of Cd-TFTPA/NH4F at 417 nm (b) and 533 nm (d), different at mospheric environments (e), the delayed lifetime decay profiles of Cd-TFTPA/NH4F at 417 nm (f).

**Figure S19.** Delayed PL spectra of Cd-TFTPA/NH_4_F detected in different pH environments (a, c), the delayed lifetime decay profiles of Cd-TFTPA/NH4F at 417 nm (b) and 525 nm (d) under different PH.

**Figure S20.** PXRD patterns of Cd-TFTPA/NH_4_F under different PH.

**Figure S21.** The simulated (black) and as-synthesized PXRD patterns for Cd-TFTPA/NH_4_F (red), Cd/Mn-TFTPA/NH_4_F (green), Cd/Pb-TFTPA/NH_4_F (blue).

**Figure S22.** Scanning electron microscopy (SEM) and Energy-dispersive X-ray spectrometry (EDX) mapping for corresponding elemental distributions in Cd/Mn-TFTPA/NH_4_F.

**Figure S23.** Scanning electron microscopy (SEM) and Energy-dispersive X-ray spectrometry (EDX) mapping for corresponding elemental distributions in Cd/Pb-TFTPA/NH_4_F.

**Figure S24.** X-ray photoelectron spectroscopy (XPS) of Cd/Mn-TFTPA/NH_4_F.

**Figure S25.** X-ray photoelectron spectroscopy (XPS) of Cd/Pb-TFTPA/NH_4_F.

**Figure S26.** Fluorescence spectra (a) and delayed PL spectra (b) of Cd/Mn-TFTPA/NH_4_F.

**Figure S27.** Fluorescence spectra of Cd/Pb-TFTPA/NH_4_F.

**Figure S28.** Schematic diagram for the energy levels of Cd/Mn-TFTPA/NH_4_F.

**Figure S29.** Schematic diagram for the energy levels of Cd/Pb-TFTPA/NH_4_F.

**Figure S30.** Photoluminescence characterization of Cd/Pb-TFTPA/NH_4_F powder under ambient conditions.

**Figure S31.** Fluorescent (a) and phosphorescence (b) Commission Internationale de l’Eclairage (CIE) diagram of sample Cd/Mn-TFTPA/NH_4_F under different excitation wavelengths.

**Figure S32.** Fluorescent (a) and phosphorescence (b) Commission Internationale de l’Eclairage (CIE) diagram of sample Cd/Pb-TFTPA/NH_4_F under different excitation wavelengths.

**Figure S33.** The JCPDS (#11-0287, black) and as-synthesized PXRD patterns for Zn-TFTPA (green), Zn-TFTPA/NH_4_F (red).

**Figure S34.** X-ray photoelectron spectroscopy (XPS) of Zn-TFTPA.

**Figure S35.** X-ray photoelectron spectroscopy (XPS) of Zn-TFTPA/NH_4_F.

**Figure S36.** Scanning electron microscopy (SEM) and Energy-dispersive X-ray spectrometry (EDX) mapping for corresponding elemental distributions in Zn-TFTPA.

**Figure S37.** Scanning electron microscopy (SEM) and Energy-dispersive X-ray spectrometry (EDX) mapping for corresponding elemental distributions in Zn-TFTPA/NH_4_F.

**Figure S38.** Photoluminescence characterization of Zn-TFTPA and Zn-TFTPA/NH_4_F powder under ambient conditions.

**Figure S39.** Fluorescence spectra (a), delayed PL spectra (b) and decay curves (c,d) of Zn-TFTPA at 460 nm and 522 nm.

**Figure S40.** Fluorescence spectra (a), delayed PL spectra (b) and decay curves (c,d) of Zn-TFTPA/NH_4_F at 465 nm and 530 nm.

**Table S1.** Phosphorescence lifetimes (τ) of Cd-TFTPA, Cd-TFTPA/NH_4_F, Cd/Mn-TFTPA/NH_4_F, Cd/Pb-TFTPA/NH_4_F, Zn-TFTPA, Zn-TFTPA/NH_4_F.


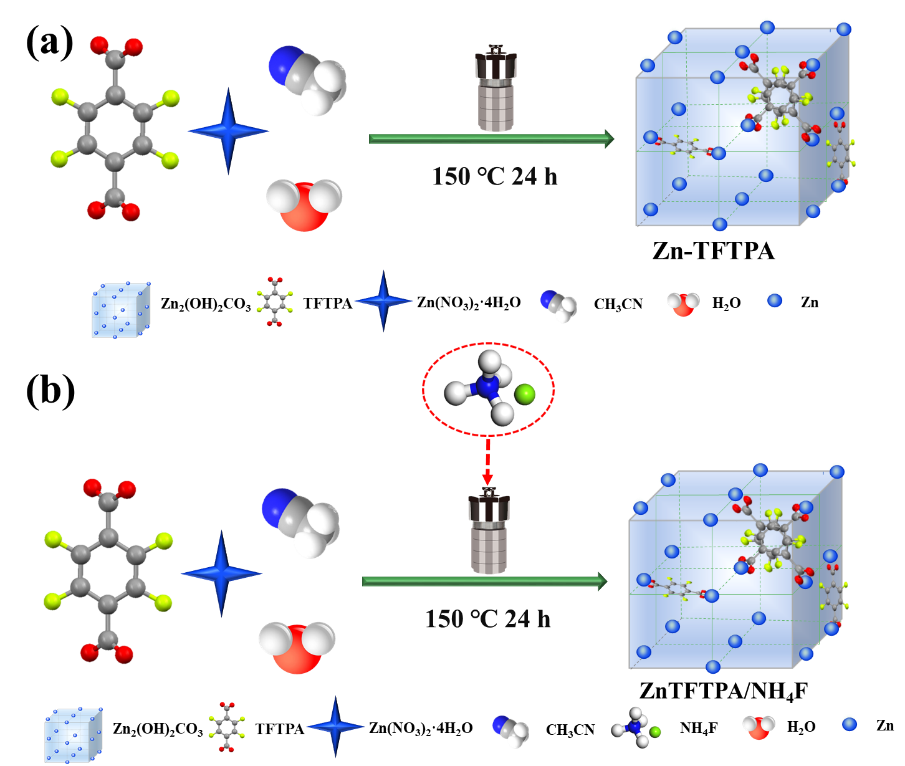


**Scheme S1.** Synthetic routes to Zn-TFTPA (a), Zn-TFTPA/NH_4_F (b).


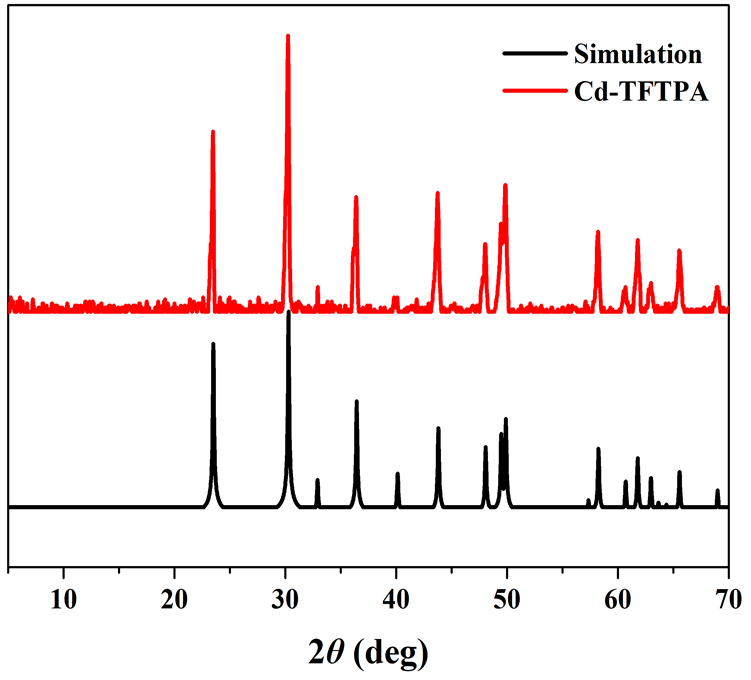


**Figure S1.** The simulated (black) and as-synthesized (red) PXRD patterns for Cd-TFTPA.


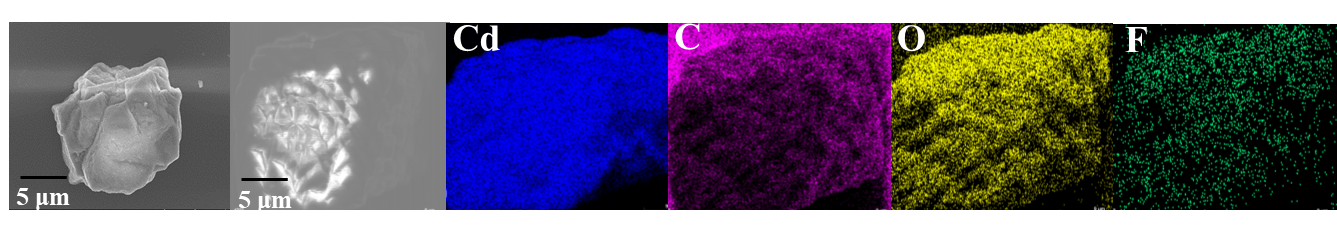


**Figure S2.** Scanning electron microscopy (SEM) and Energy-dispersive X-ray spectrometry (EDX) mapping for corresponding elemental distributions in Cd-TFTPA.


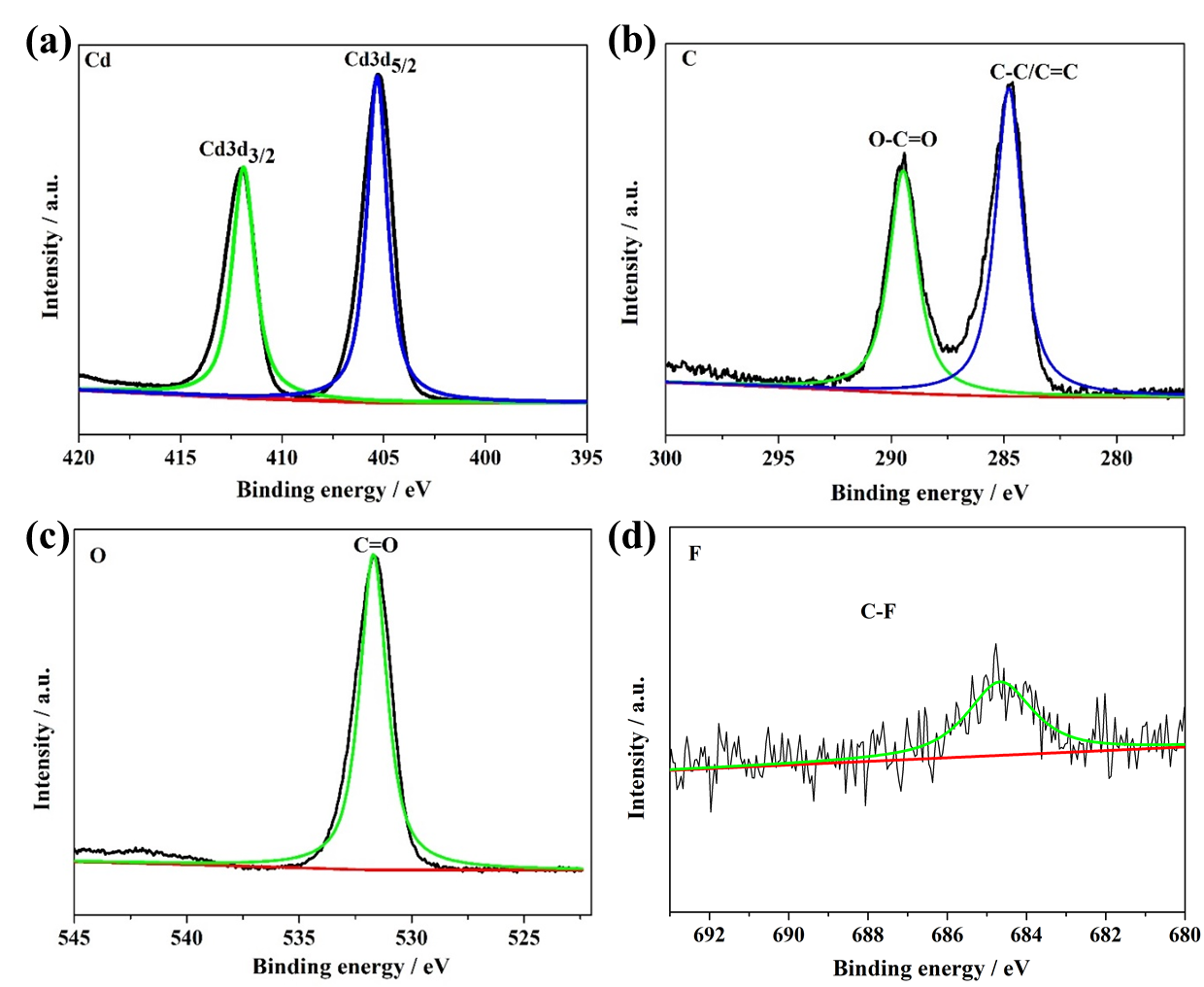


**Figure S3.** X-ray photoelectron spectroscopy (XPS) of Cd-TFTPA.





**Figure S4.** Fluorescence spectra of Cd-TFTPA.


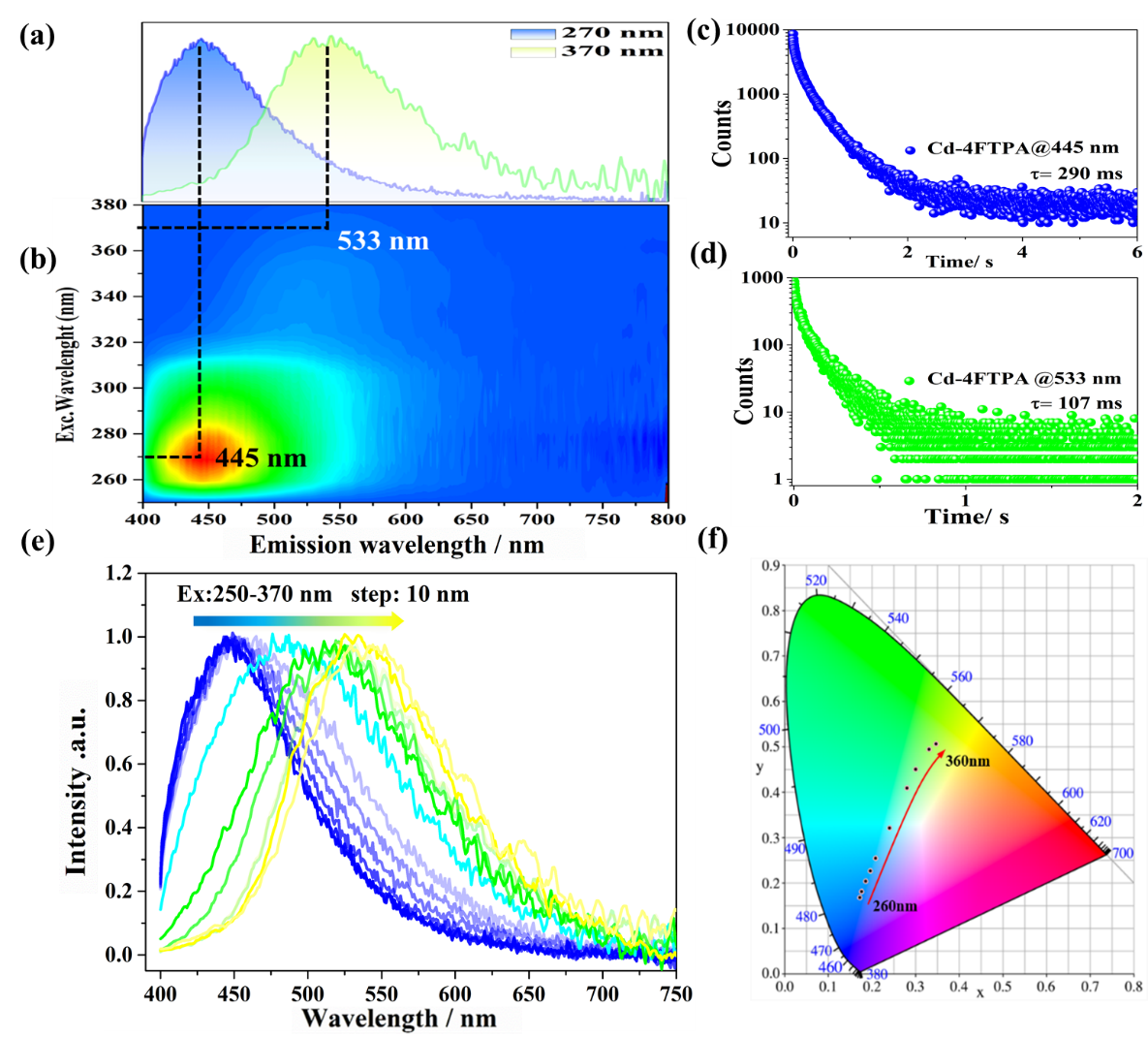


**Figure S5.** Photoluminescence characterization of Cd-TFTPA powder under ambient conditions. a) The URTP spectra of the Cd-TFTPA powder under the excitation at 270 nm (blue) and 370 nm (green), respectively. b) Excitation–phosphorescence mapping of powder under ambient conditions. c,d) decay curves of Cd-TFTPA at 445 nm and 533 nm. e) Excitation dependent phosphorescence spectras of Cd-TFTPA. f) CIE coordinate diagram of Cd-TFTPA by changing the excitation wavelengths.


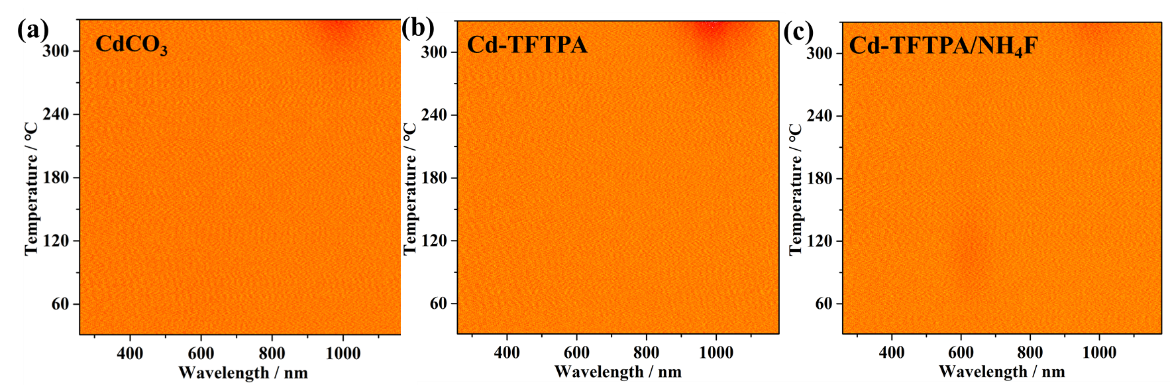


**Figure S6.** The thermostimulated luminescence (TSL) text of pure CdCO_3_ (a), Cd-4FTPA (b) and Cd-TFTPA/NH_4_F (c).


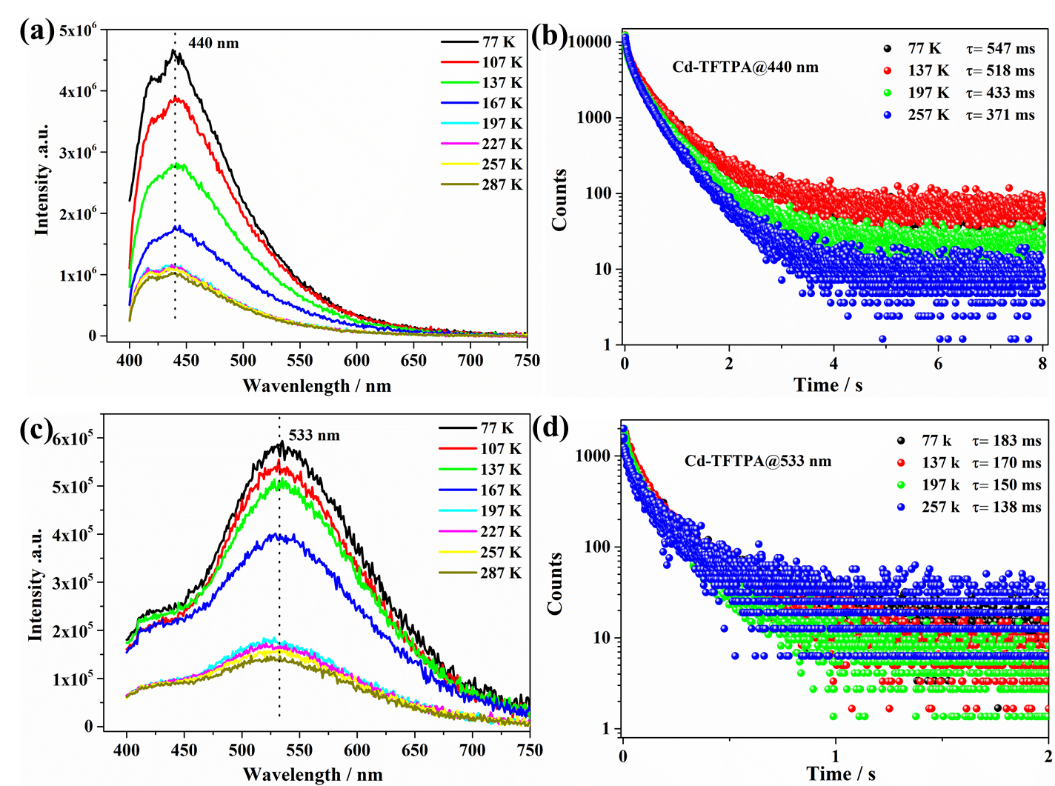


**Figure S7.** The temperature-dependent delayed PL spectra (a,c) and time-resolved PL-decay profiles of Cd-TFTPA under different temperature at 440 nm and 533 nm.





**Figure S8.** Phosphorescence spectra of TFTPA in a dilute solution of Tetrahydrofuran (THF) under 280, 320 and 350 nm excitation at 77 K.


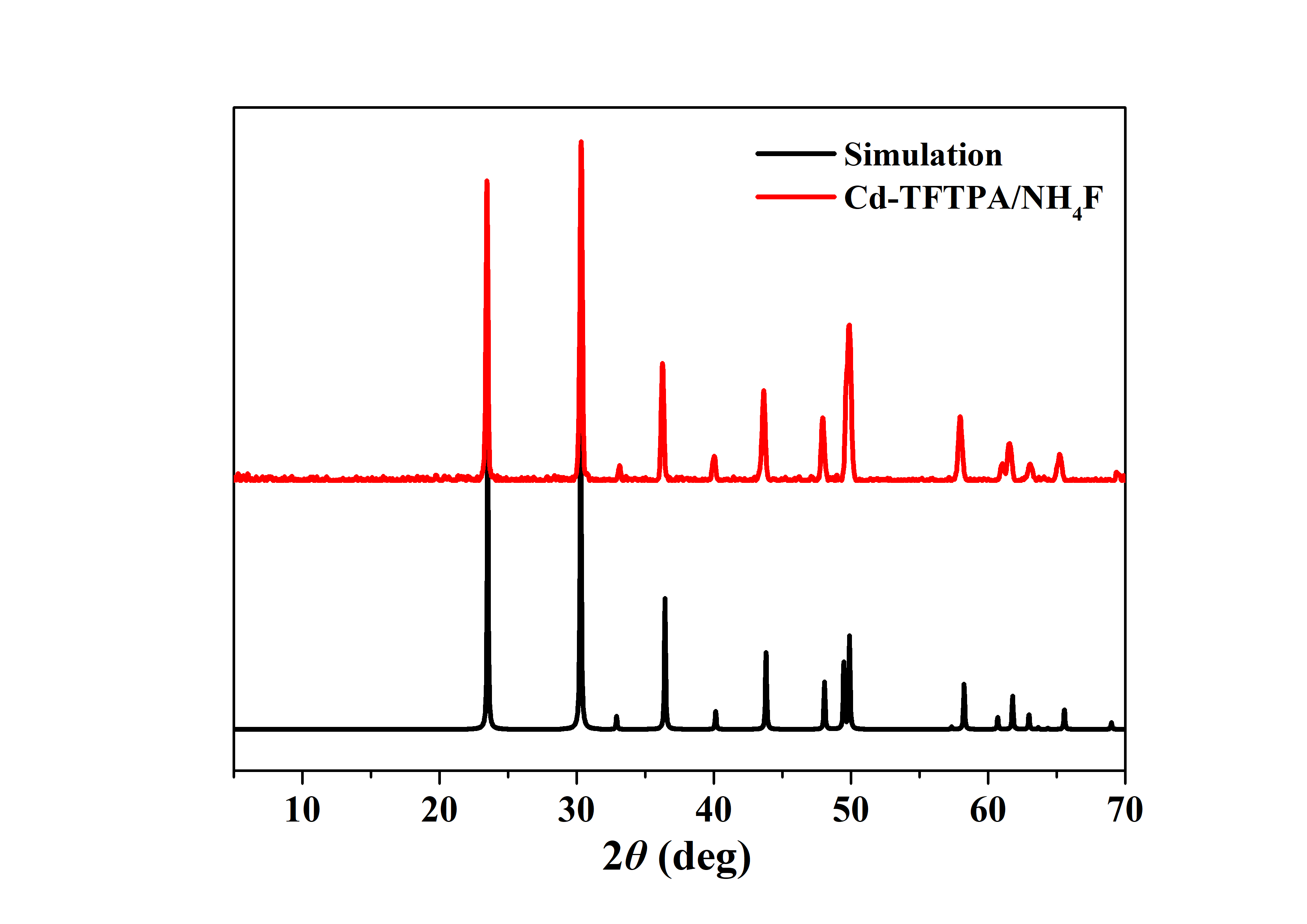


**Figure S9.** The simulated (black) and as-synthesized (red) PXRD patterns for Cd-TFTPA/NH_4_F.


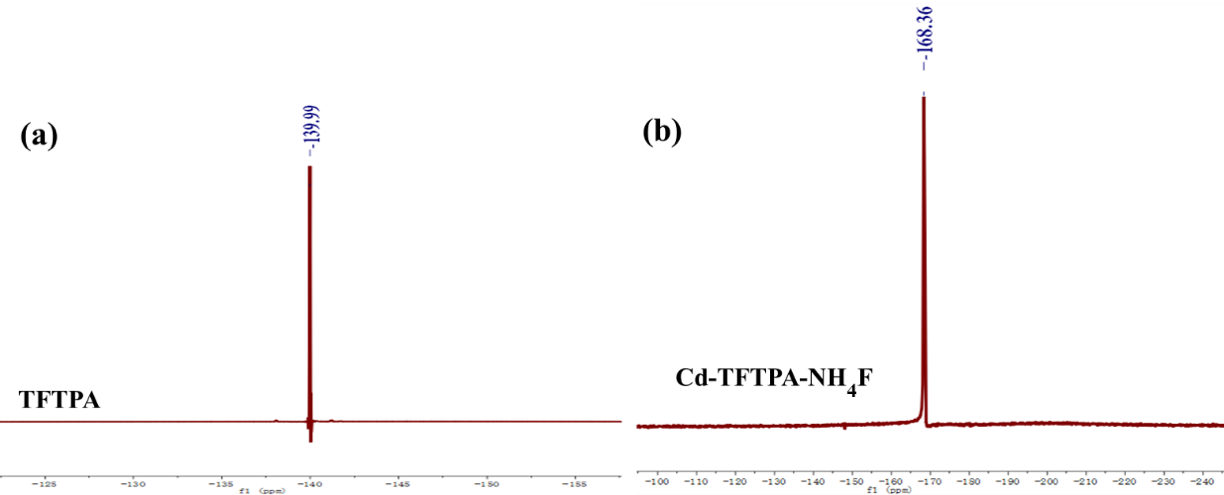


**Figure S10.** ^19^FNMR of TFTPA (a) and Cd-TFTPA/NH_4_F (b).


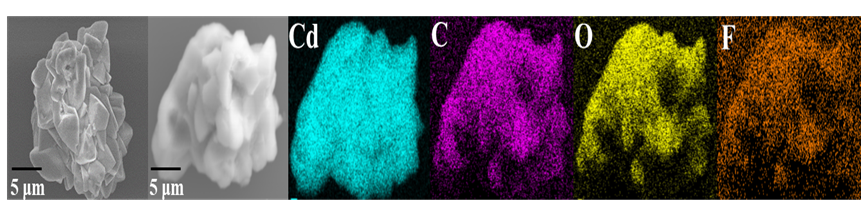


**Figure S11.** Scanning electron microscopy (SEM) and Energy-dispersive X-ray spectrometry (EDX) mapping for corresponding elemental distributions in Cd-TFTPA/NH_4_F.


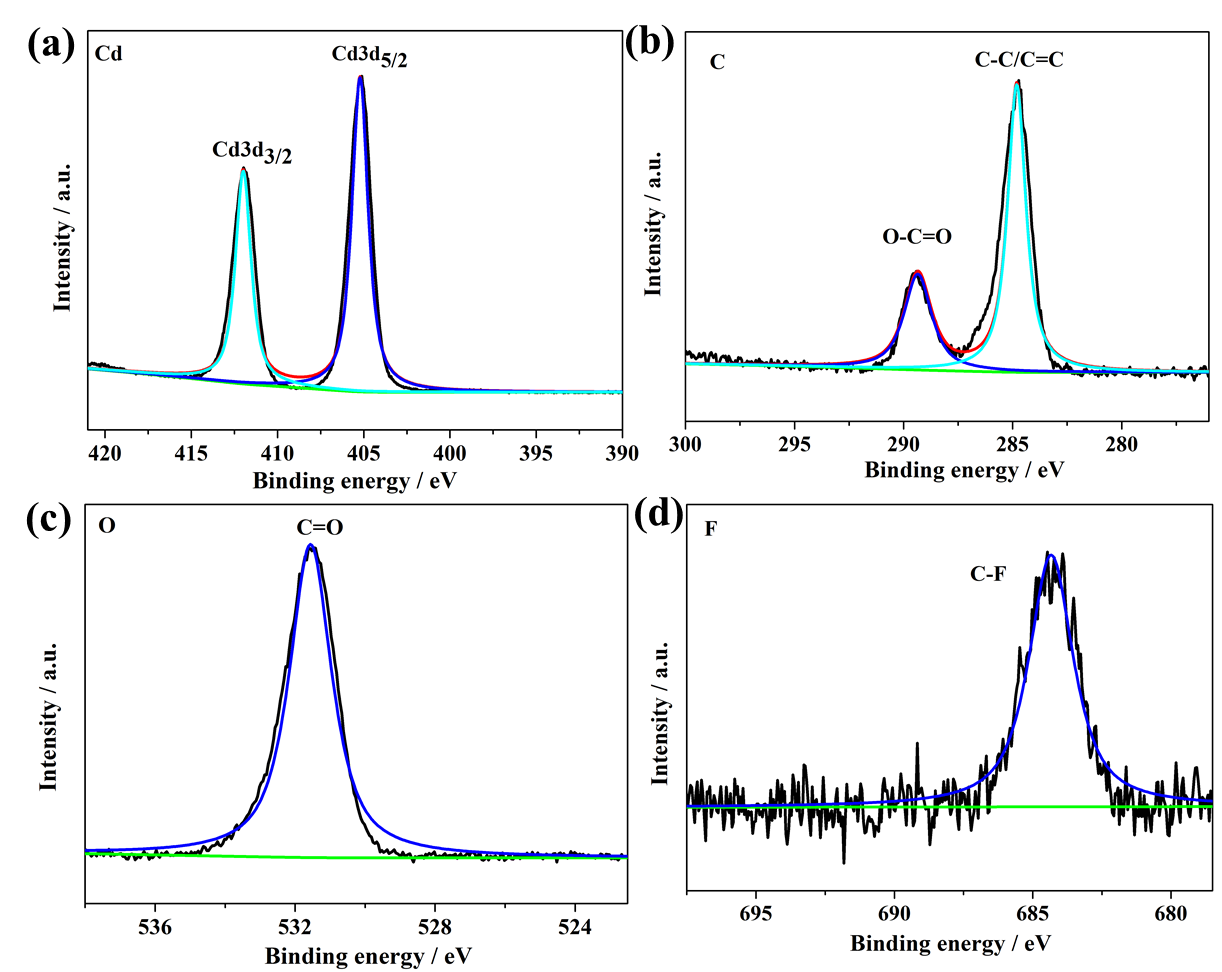


**Figure S12.** X-ray photoelectron spectroscopy (XPS) of Cd-TFTPA/NH_4_F.


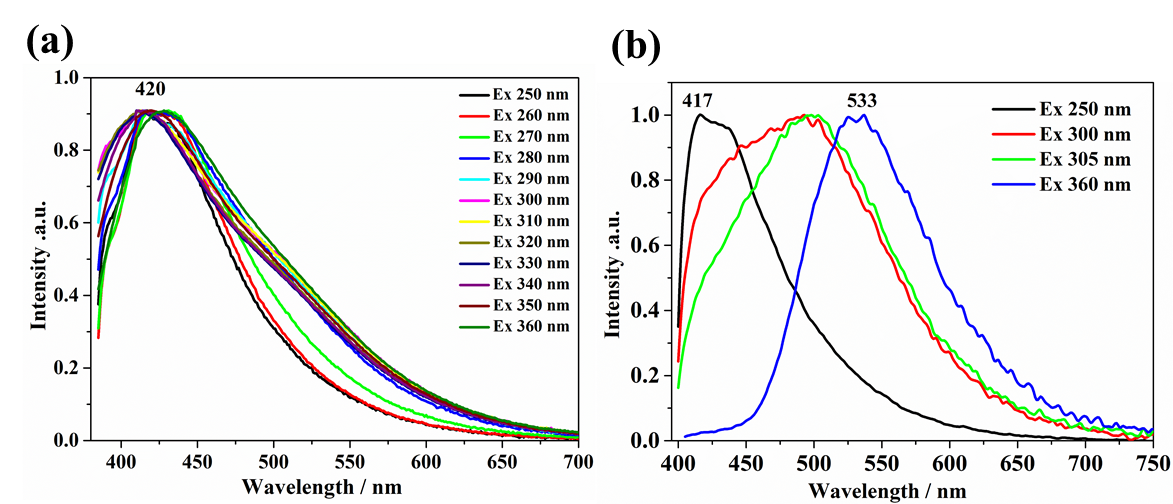


**Figure S13.** Fluorescence spectra (a) and delayed PL spectra (b) of Cd-TFTPA/NH_4_F.


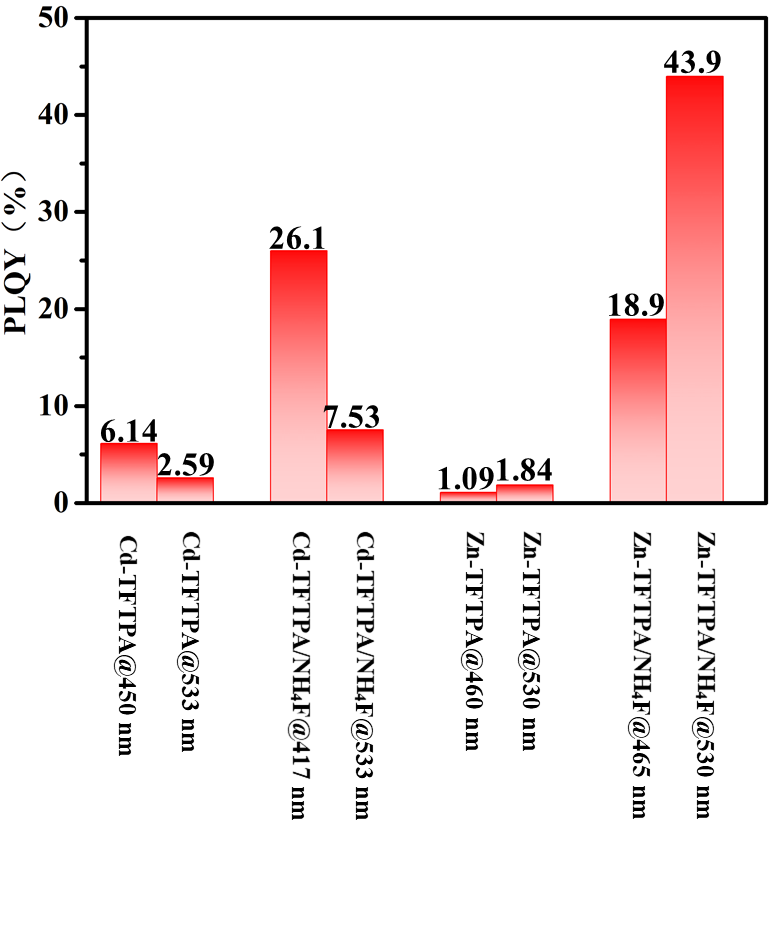


**Figure S14.** The comparison of PLQY values for Cd-TFTPA, Zn-TFTPA, Cd-TFTPA/NH_4_F, Zn-TFTPA/NH_4_F under different wavelengths.


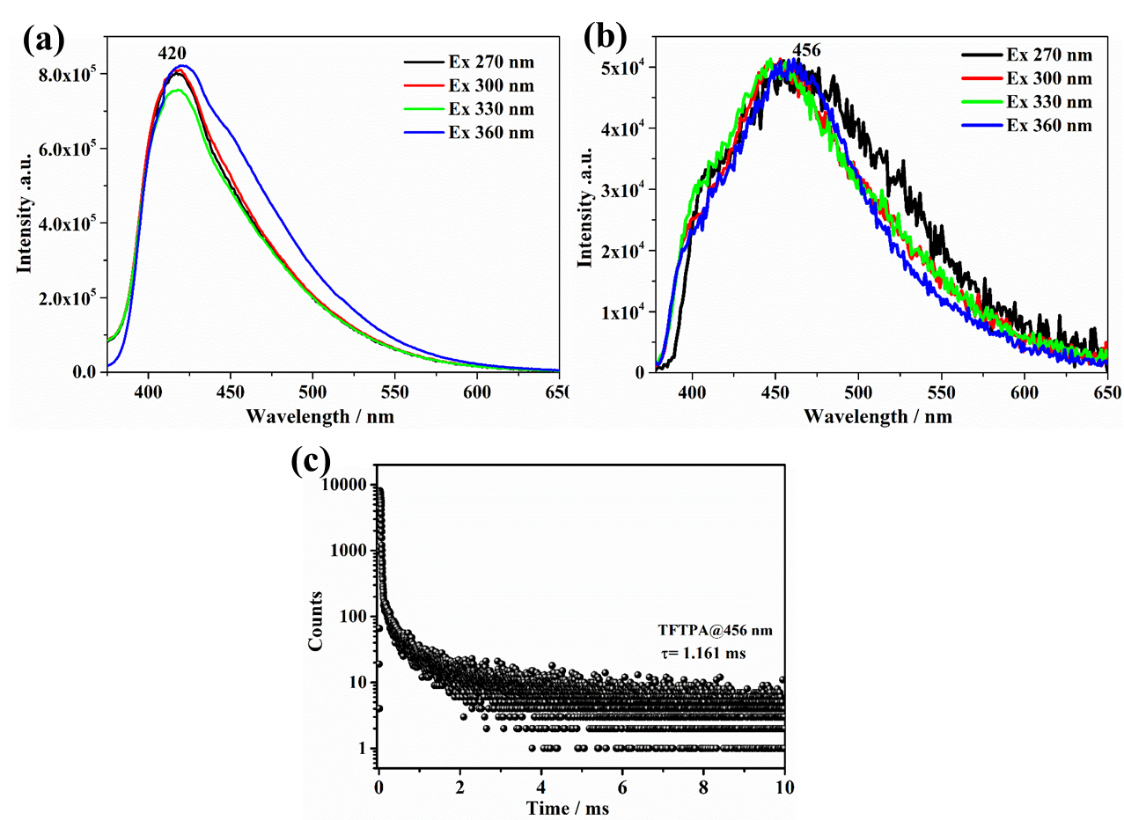


**Figure S15.** Fluorescence spectra (a), delayed PL spectra (b) and decay curves (c) of TFTPA at 456 nm.


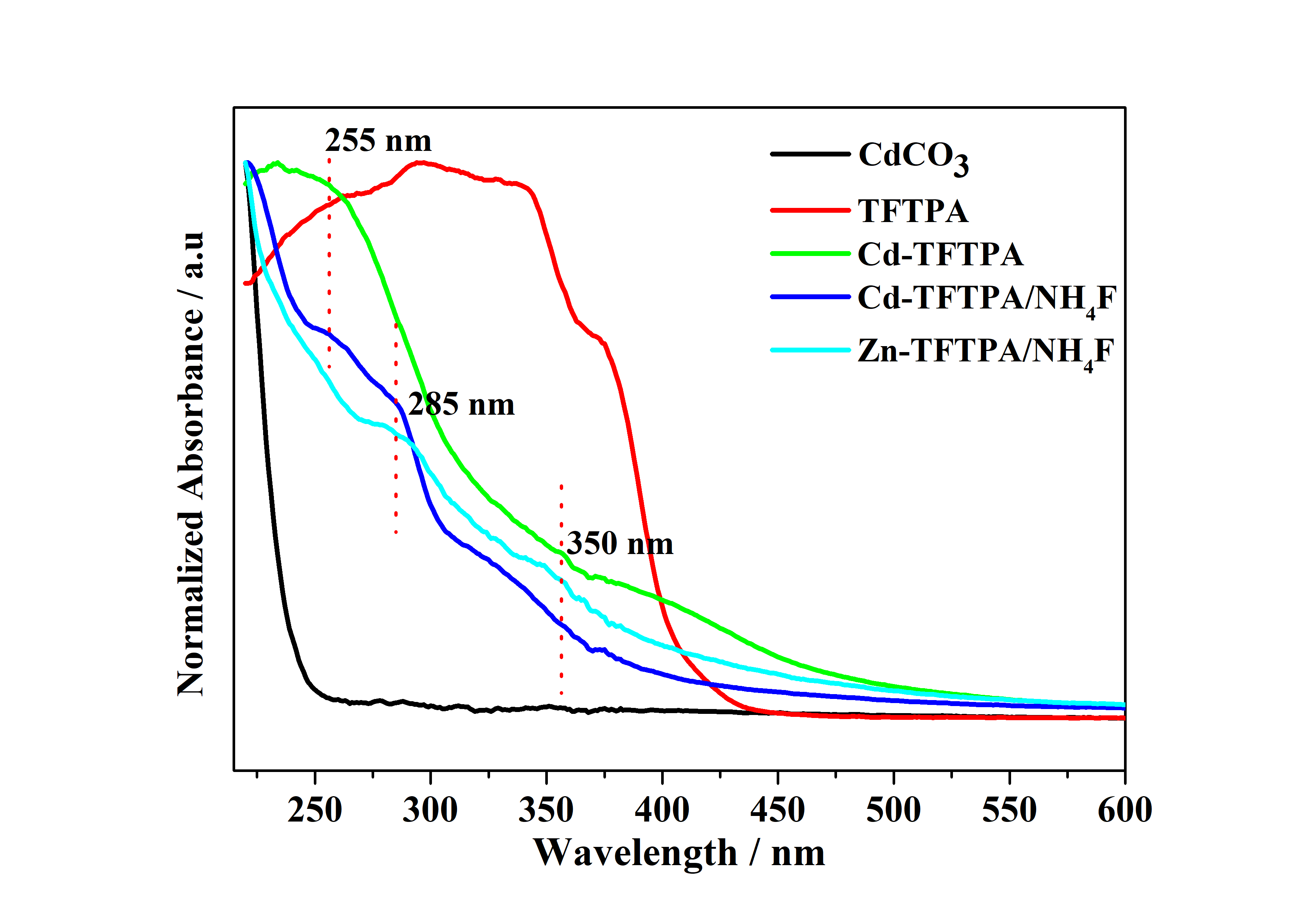


**Figure S16.** Solid-state UV-vis absorption spectra of CdCO_3_, TFTPA and Cd-TFTPA, Cd-TFTPA/NH_4_F, Zn-TFTPA/NH_4_F under ambient conditions.


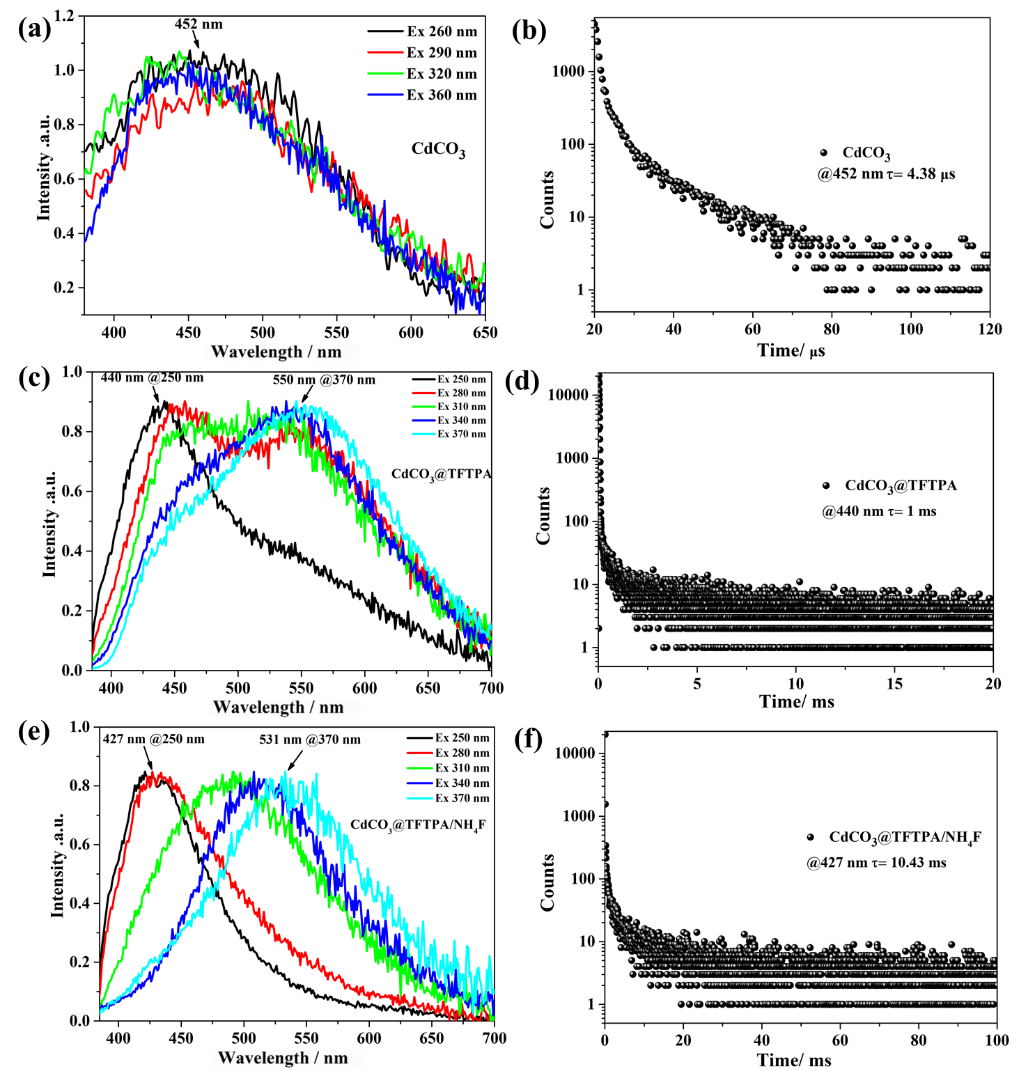


**Figure S17.** The delayed PL spectra of CdCO_3_ (a) CdCO_3_@TFTPA (b) CdCO_3_@TFTPA/NH_4_F (c), the delayed lifetime decay profiles at 452 nm of CdCO_3_ (b), 440 nm of CdCO_3_@TFTPA (d) and 427 nm of CdCO_3_@TFTPA/NH_4_F (f).


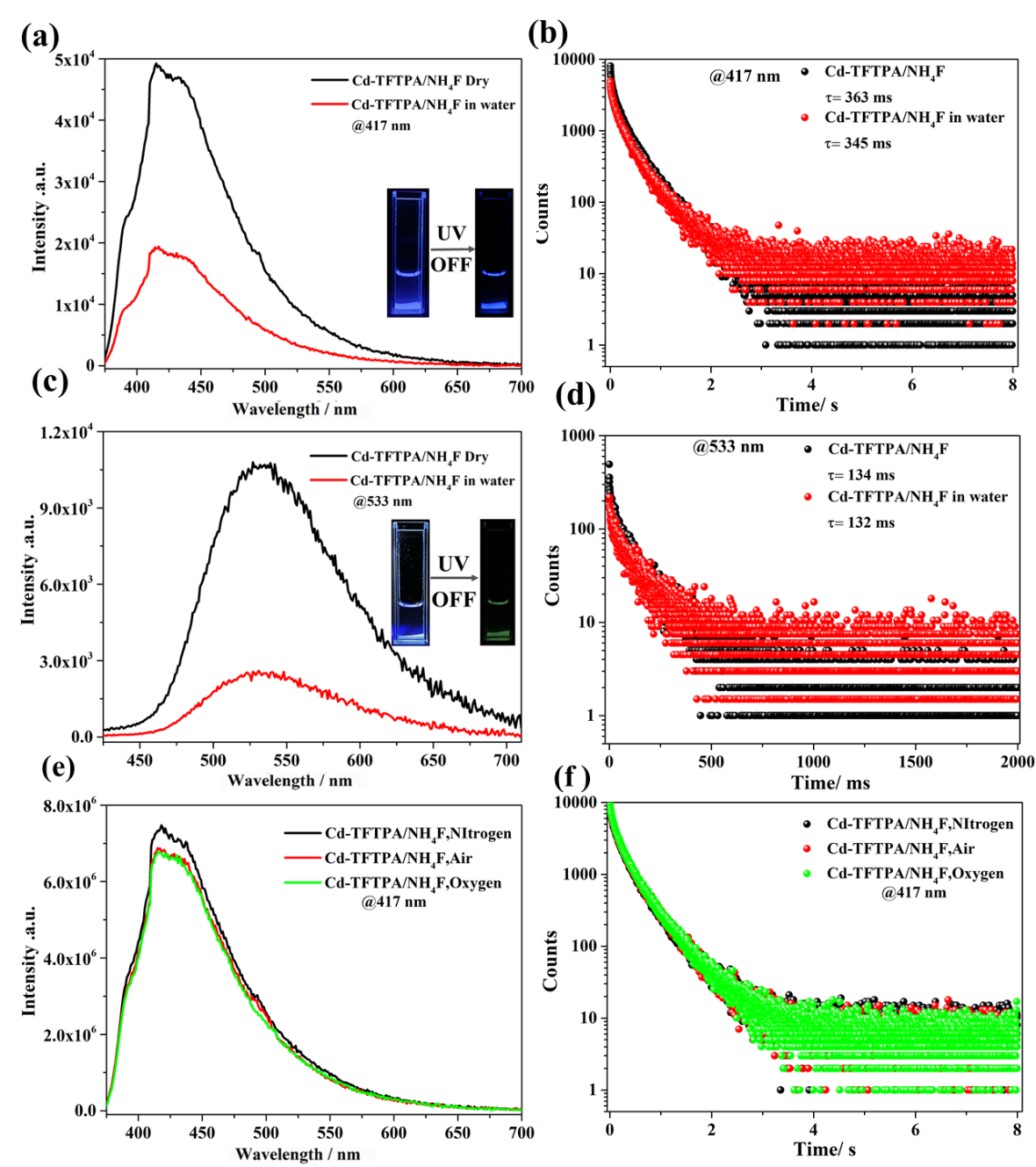


**Figure S18.** Delayed PL spectra of Cd-TFTPA/NH_4_F detected in wet and dry conditions (a, c), the delayed lifetime decay profiles of Cd-TFTPA/NH_4_F at 417 nm (b) and 533 nm (d), different at mospheric environments (e), the delayed lifetime decay profiles of Cd-TFTPA/NH_4_F at 417 nm (f).


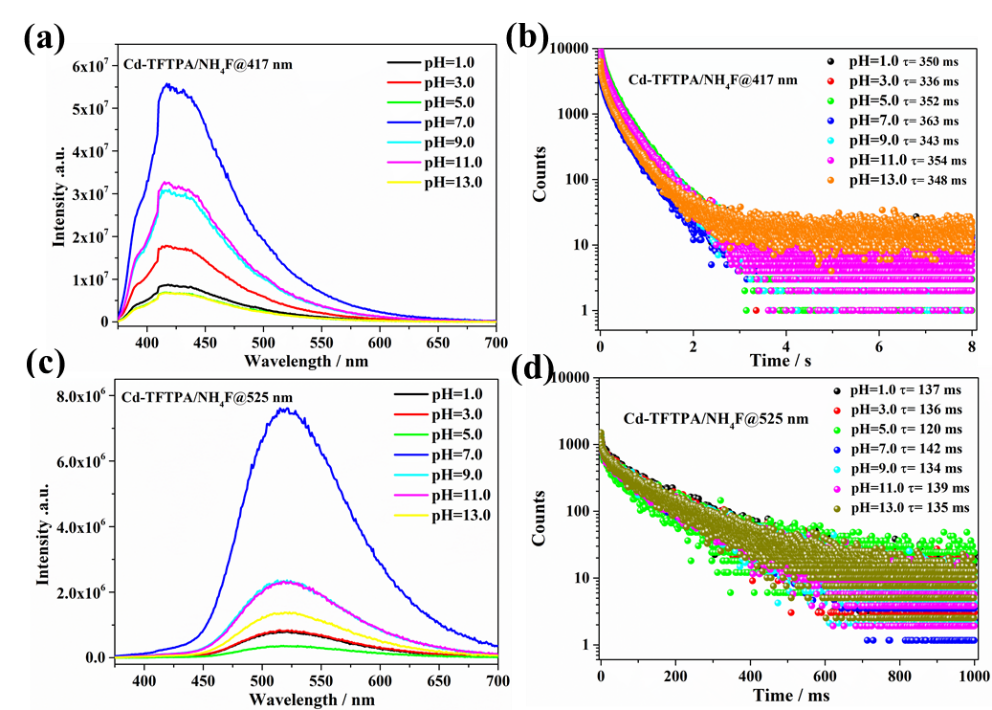


**Figure S19.** Delayed PL spectra of Cd-TFTPA/NH_4_F detected in different pH environments (a, c), the delayed lifetime decay profiles of Cd-TFTPA/NH_4_F at 417 nm (b) and 525 nm (d) under different pH.


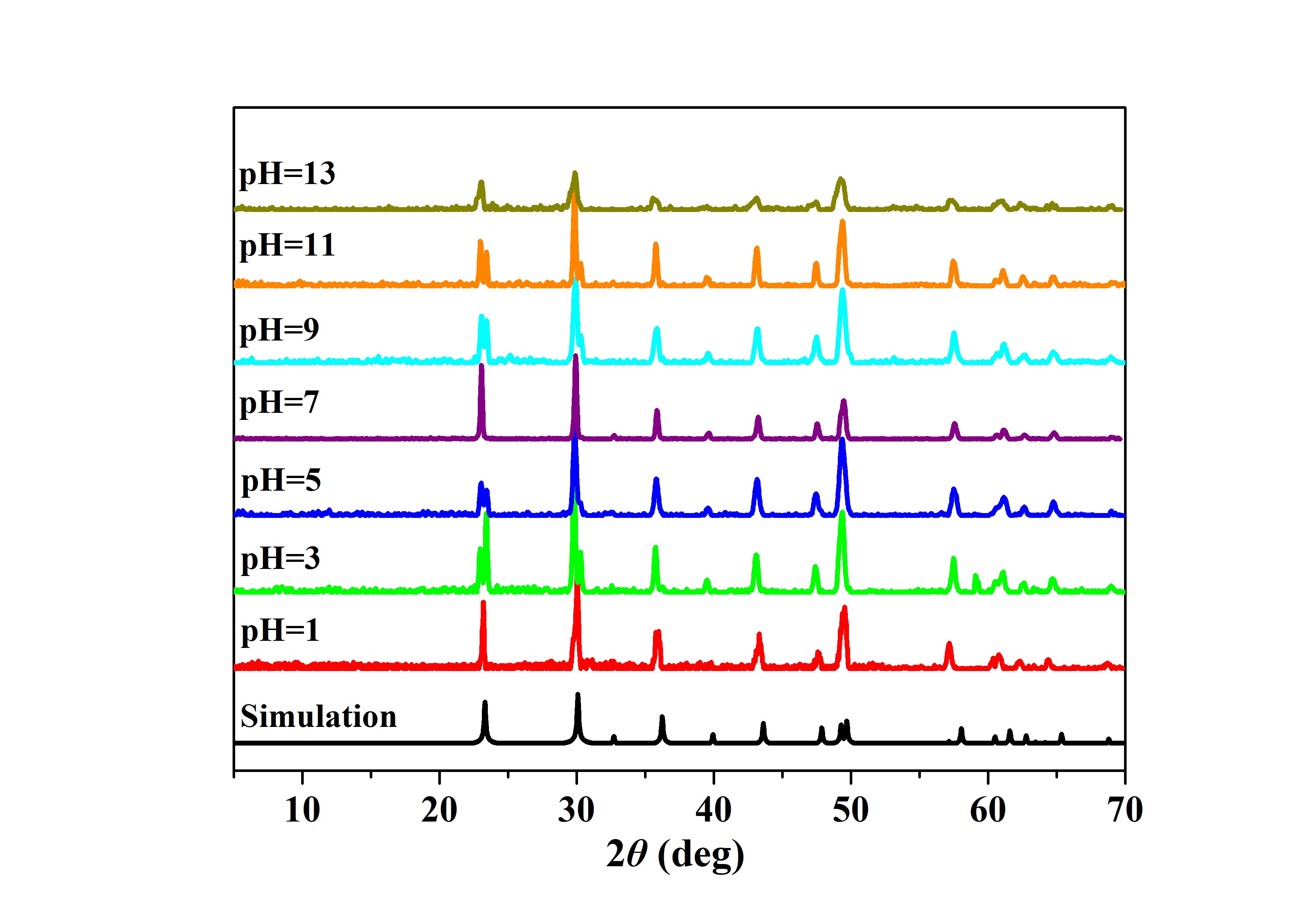


**Figure S20.** PXRD patterns of Cd-TFTPA/NH_4_F under different pH.





**Figure S21.** The simulated (black) and as-synthesized PXRD patterns for Cd-TFTPA/NH_4_F (red), Cd/Mn-TFTPA/NH_4_F (green), Cd/Pb-TFTPA/NH_4_F (blue).


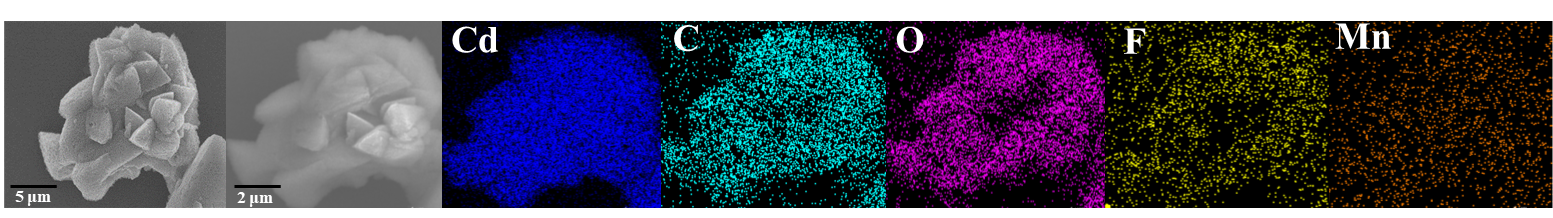


**Figure S22.** Scanning electron microscopy (SEM) and Energy-dispersive X-ray spectrometry (EDX) mapping for corresponding elemental distributions in Cd/Mn-TFTPA/NH_4_F.


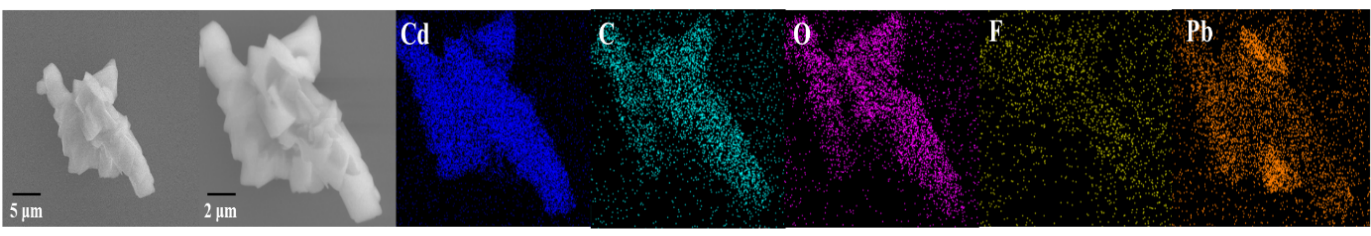


**Figure S23.** Scanning electron microscopy (SEM) and Energy-dispersive X-ray spectrometry (EDX) mapping for corresponding elemental distributions in Cd/Pb-TFTPA/NH_4_F.


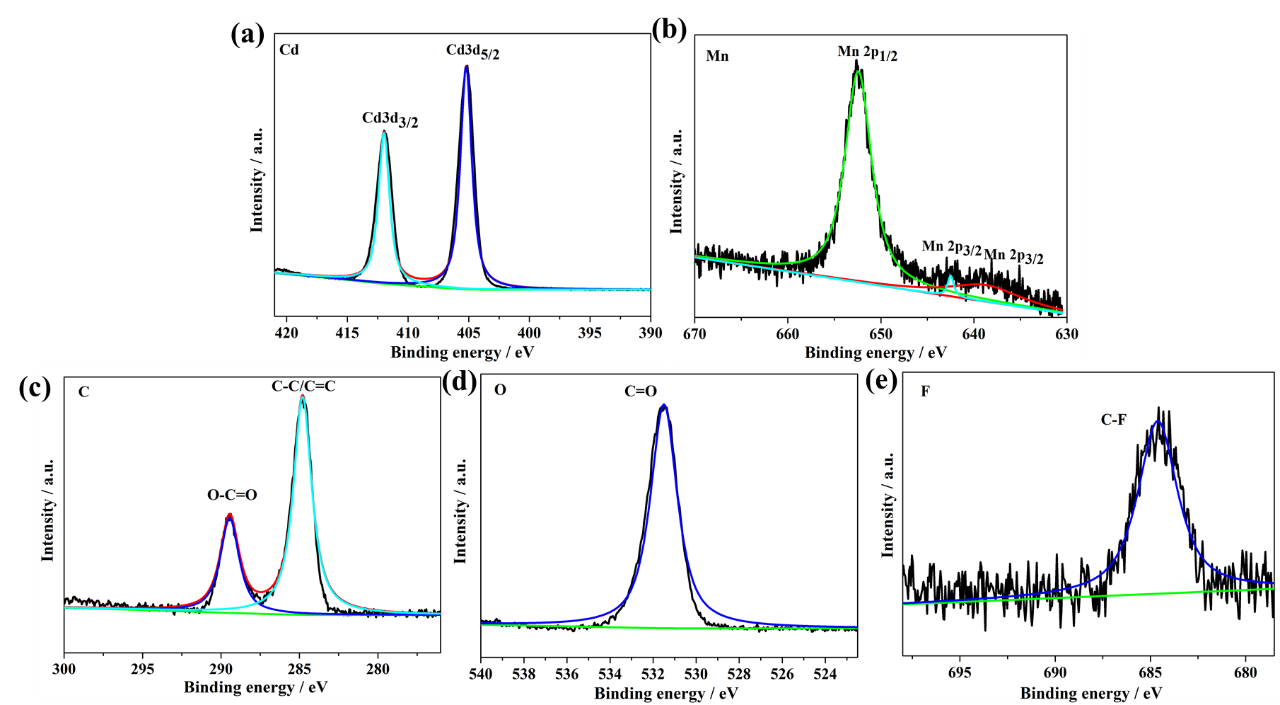


**Figure S24.** X-ray photoelectron spectroscopy (XPS) of Cd/Mn-TFTPA/NH_4_F.


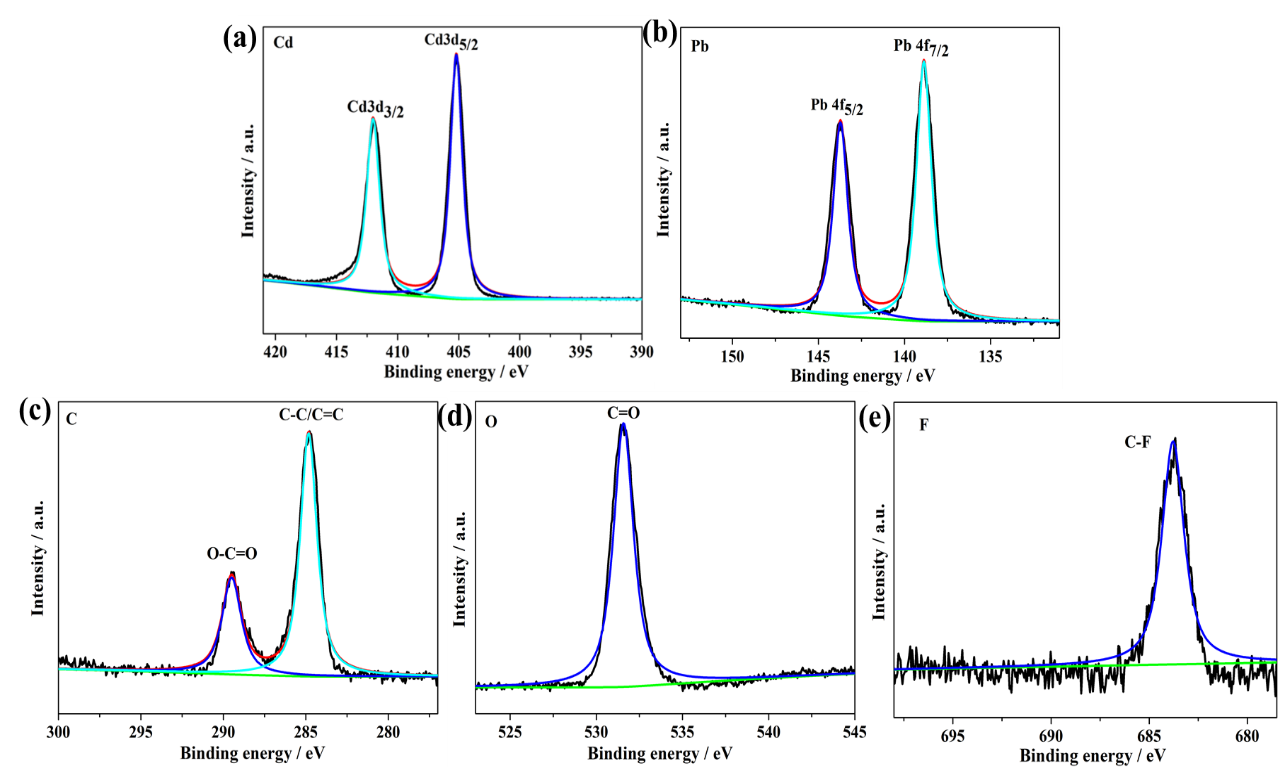


**Figure S25.** X-ray photoelectron spectroscopy (XPS) of Cd/Pb-TFTPA/NH_4_F.


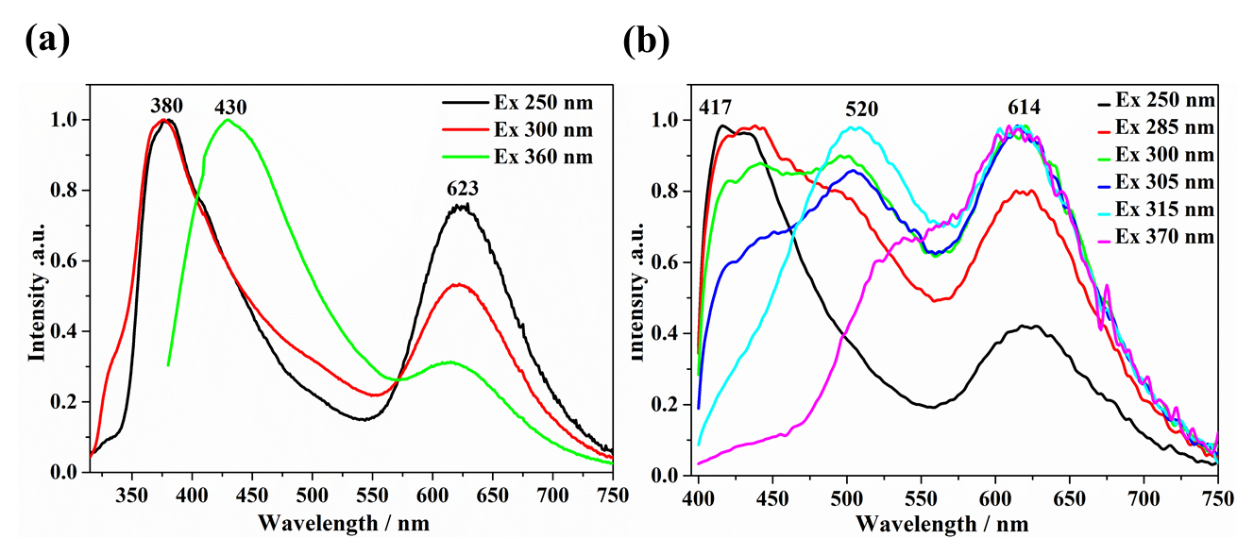


**F****igure S26.** Fluorescence spectra (a) and delayed PL spectra (b) of Cd/Mn-TFTPA/NH_4_F.


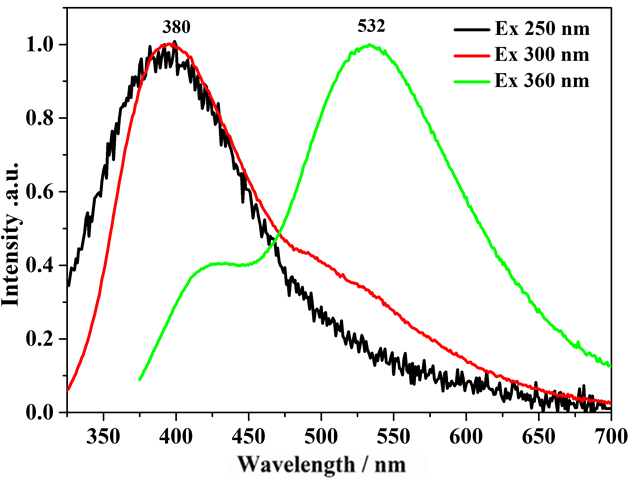


**Figure S27.** Fluorescence spectra of Cd/Pb-TFTPA/NH_4_F.


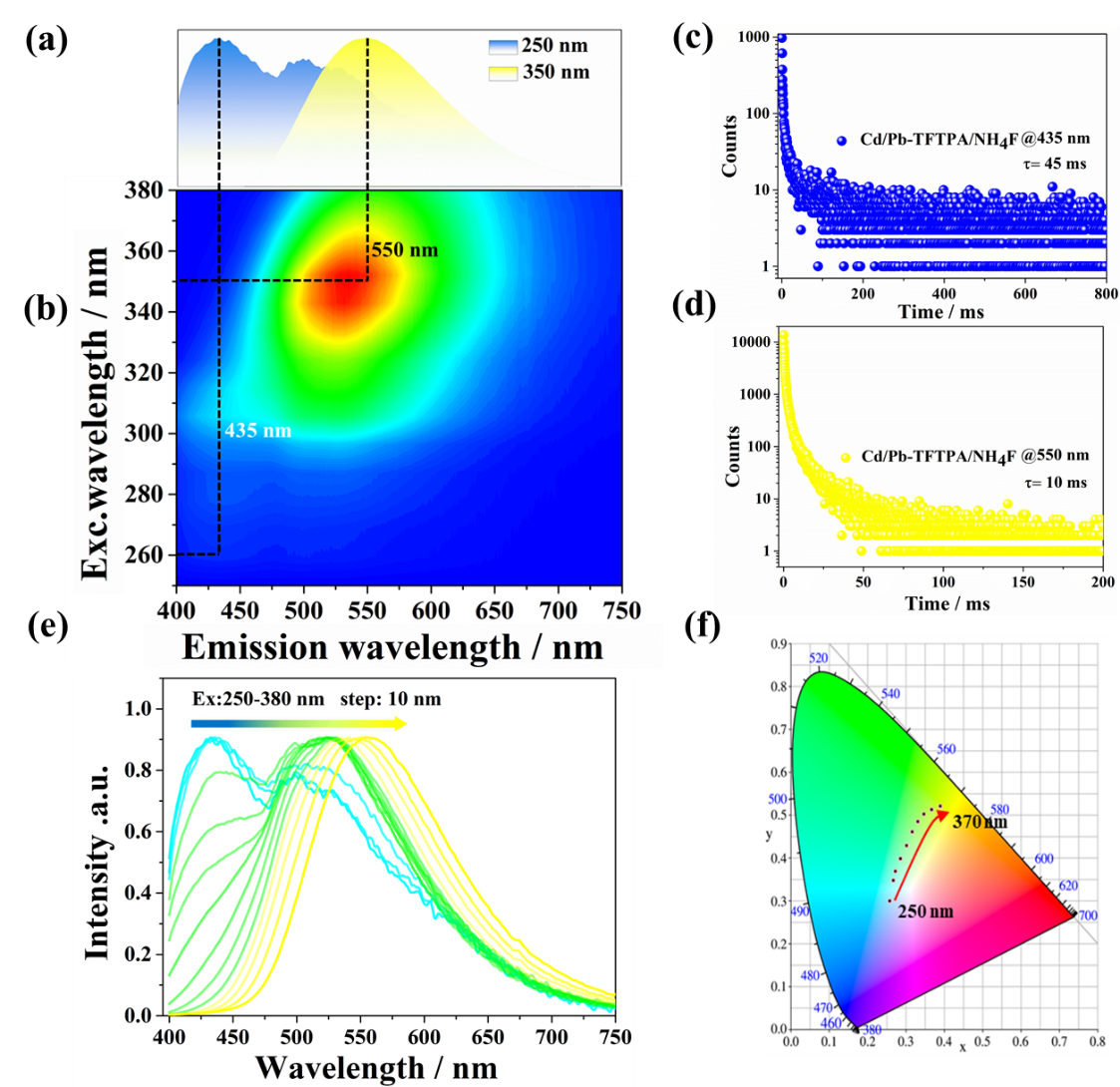


**Figure S28.** Photoluminescence characterization of Cd/Pb-TFTPA/NH_4_F powder under ambient conditions. a) The URTP spectra of the Cd/Pb-TFTPA/NH_4_F powder under the excitation at 250 nm (blue) and 350 nm (yellow), respectively. b) Excitation–phosphorescence mapping of powder under ambient conditions. c,d) decay curves of Cd/Pb-TFTPA/NH_4_F at 435 nm and 550 nm. e) Excitation dependent phosphorescence spectras of Cd/Pb-TFTPA/NH_4_F. f) CIE coordinate diagram of Cd/Pb-TFTPA/NH_4_F by changing the excitation wavelengths.


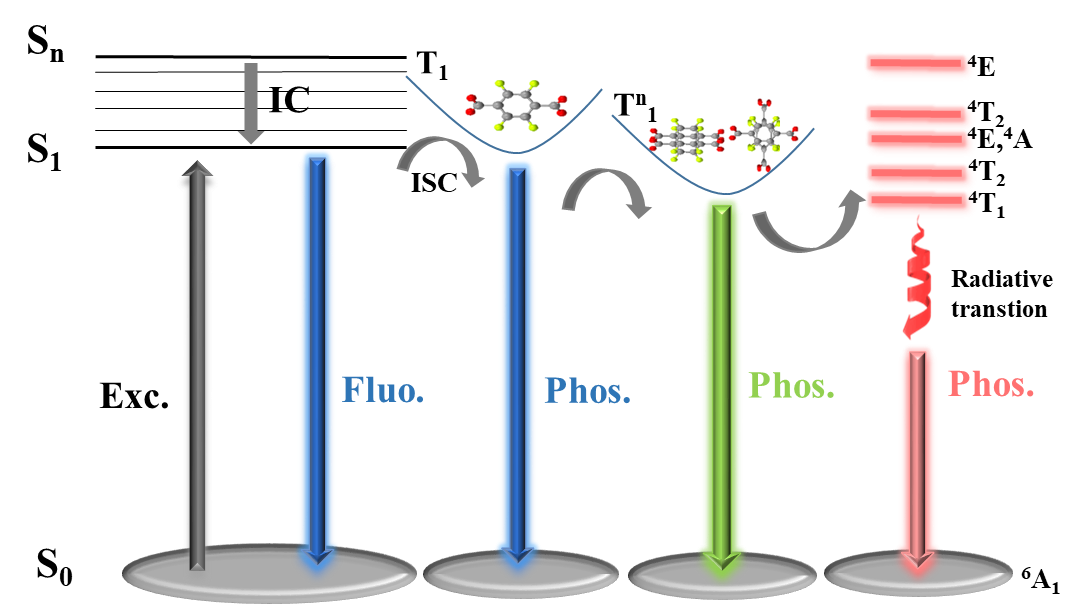


**Figure S29.** Schematic diagram for the energy levels of Cd/Mn-TFTPA/NH_4_F.


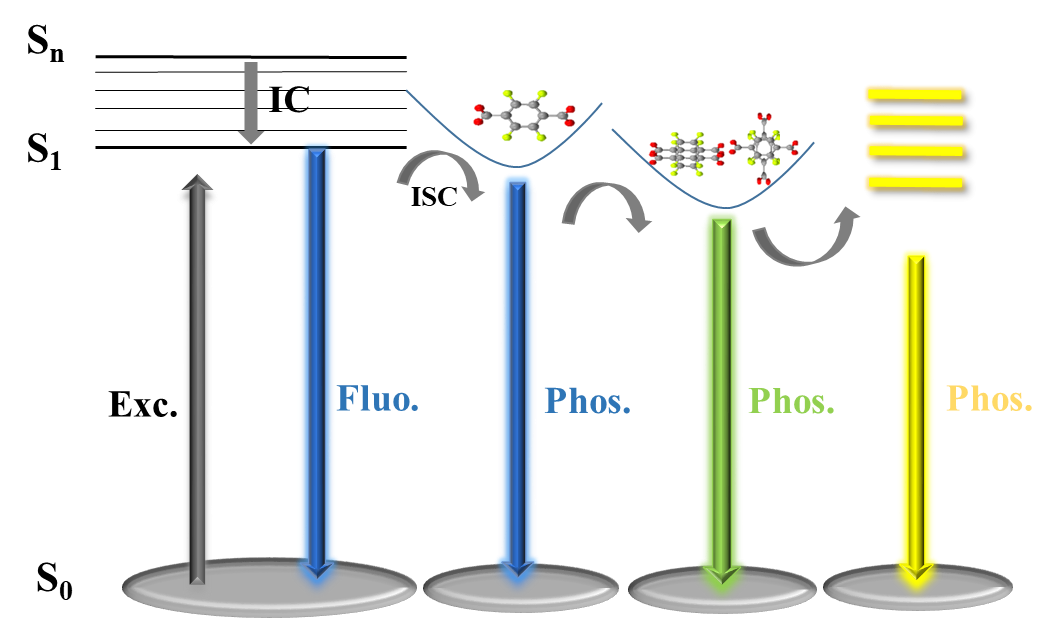


**Figure S30.** Schematic diagram for the energy levels of Cd/Pb-TFTPA/NH_4_F.


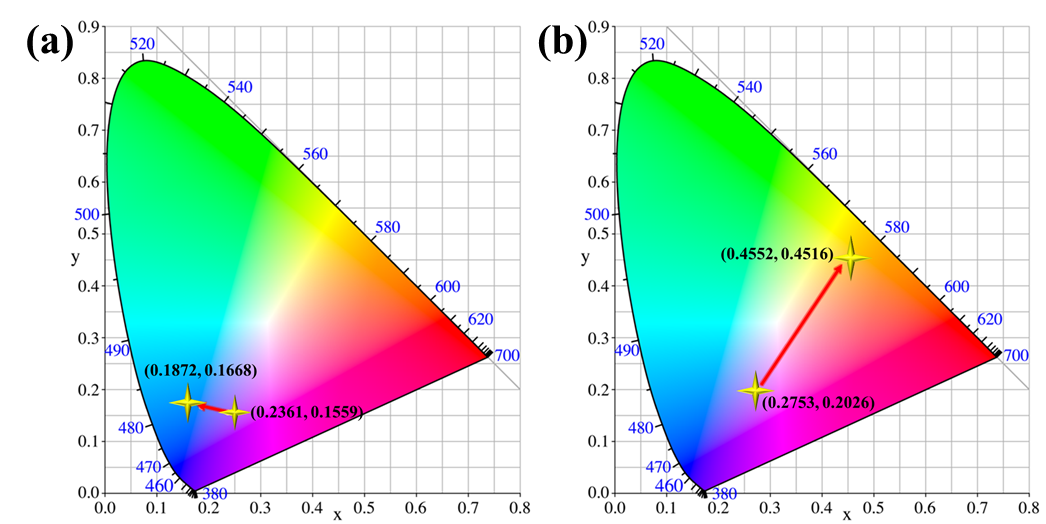


**Figure S31.** Fluorescent (a) and phosphorescence (b) Commission Internationale de l’Eclairage (CIE) diagram of sample Cd/Mn-TFTPA/NH_~~4~~_F under different excitation wavelengths.


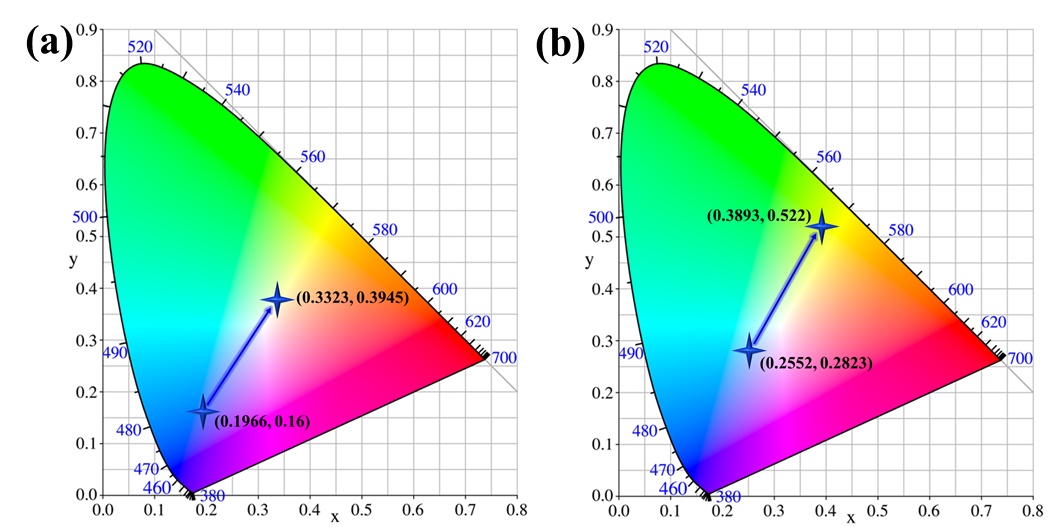


**Figure S32.** Fluorescent (a) and phosphorescence (b) Commission Internationale de l’Eclairage (CIE) diagram of sample Cd/Pb-TFTPA/NH_4_F under different excitation wavelengths.


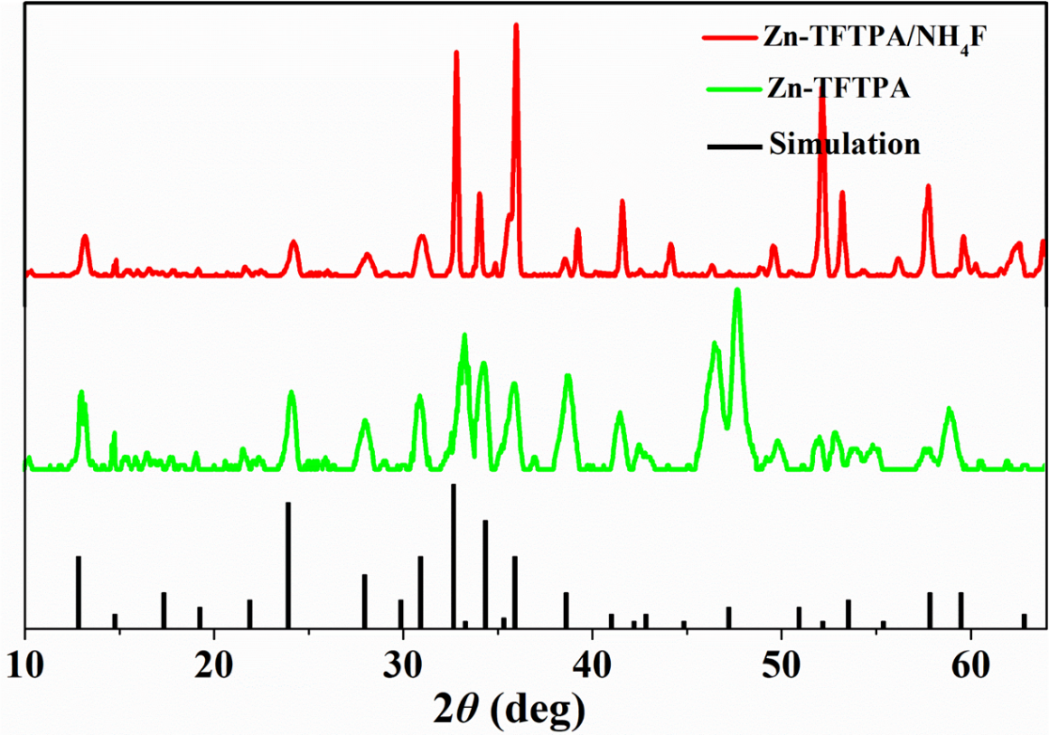


**Figure S33.** The JCPDS (#11-0287, black) and as-synthesized PXRD patterns for Zn-TFTPA (green), Zn-TFTPA/NH_4_F (red).


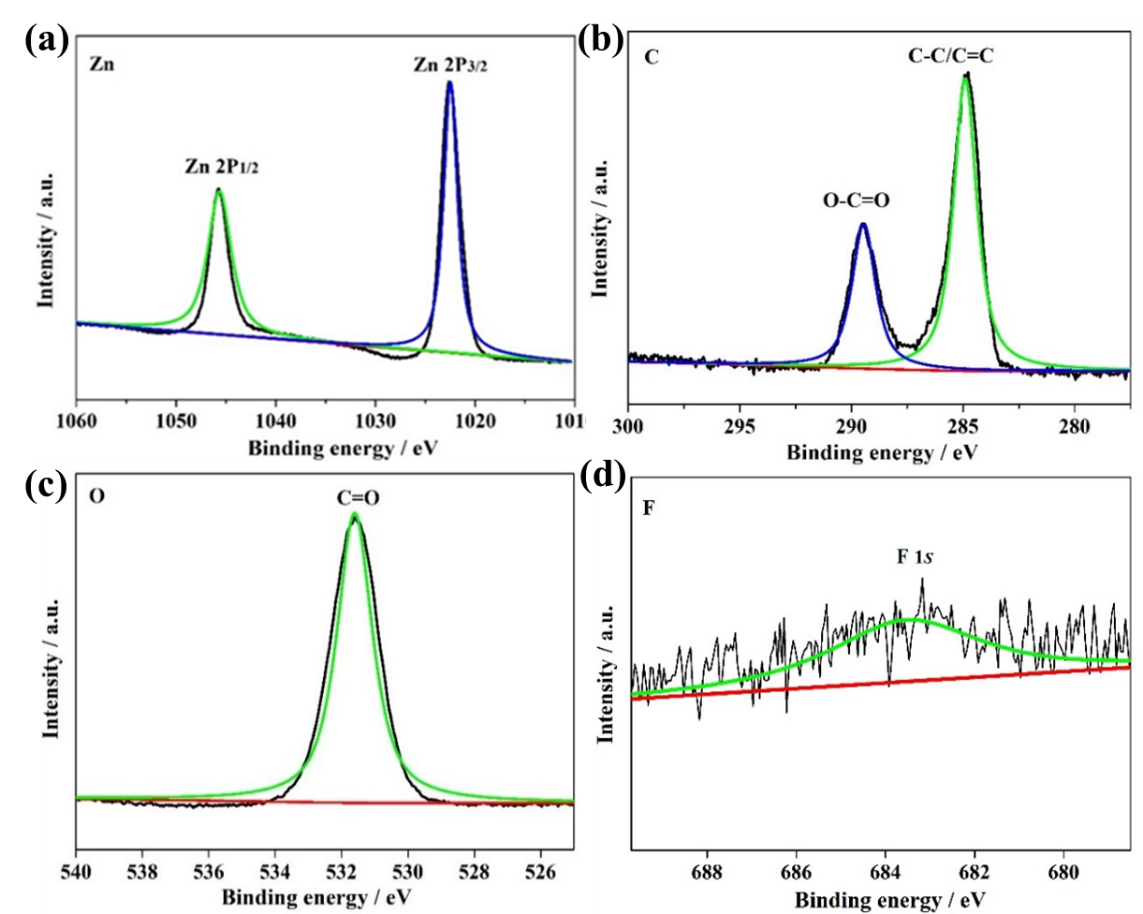


**Figure S34.** X-ray photoelectron spectroscopy (XPS) of Zn-TFTPA.


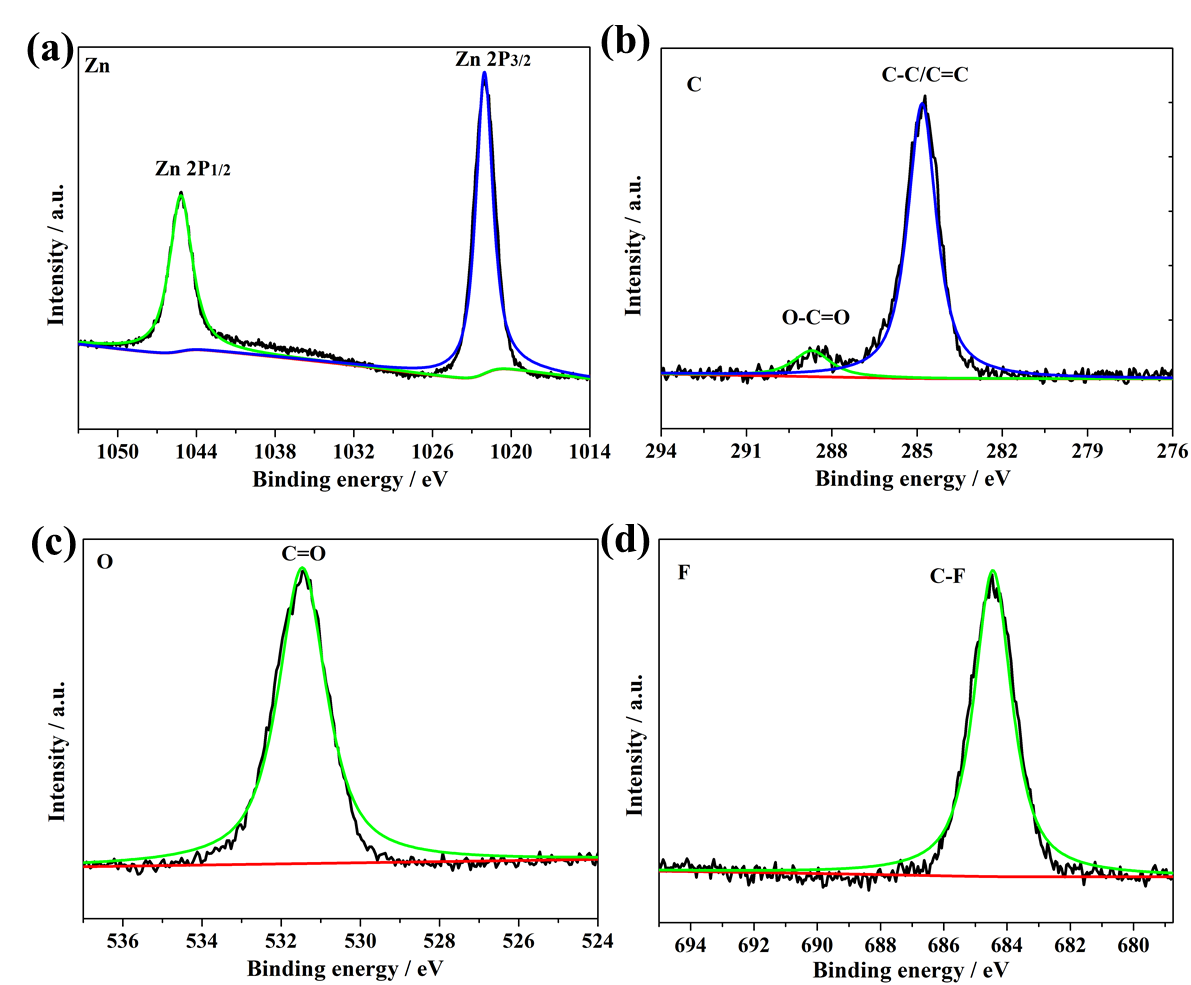


**Figure S35.** X-ray photoelectron spectroscopy (XPS) of Zn-TFTPA/NH_4_F.

The peaks centered at 1045.1 and 1022.03 eV are attributed to the Zn 2P_1/2_ and Zn 2P_3/2_ of Zn^2+^ in Zn-TFTPA, Zn-TFTPA/NH_4_F, respectively. Analysis of other elements such as C, O, F shows that TFTPA molecules were successfully introduced (**Figure S34, S35**).


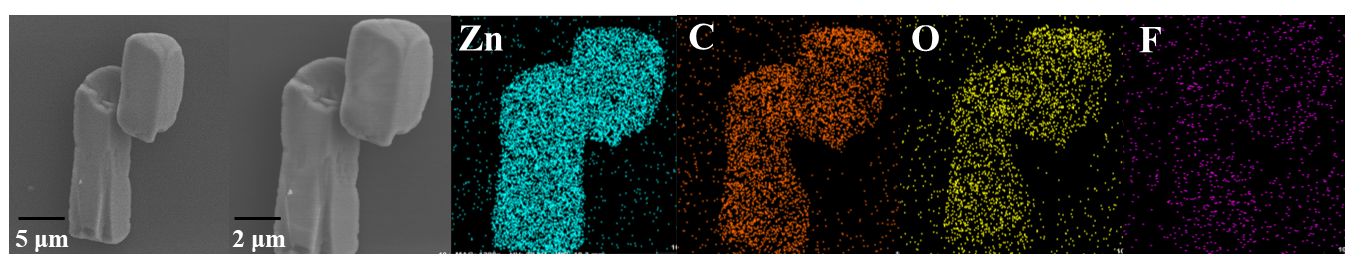


**Figure S36.** Scanning electron microscopy (SEM) and Energy-dispersive X-ray spectrometry (EDX) mapping for corresponding elemental distributions in Zn-TFTPA.


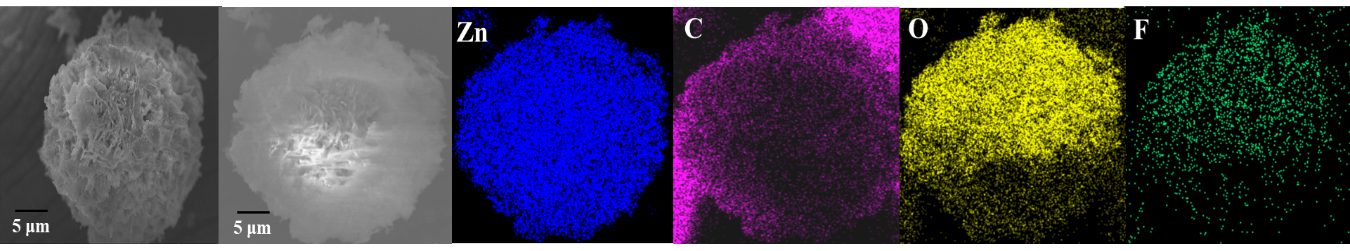


**Figure S37.** Scanning electron microscopy (SEM) and Energy-dispersive X-ray spectrometry (EDX) mapping for corresponding elemental distributions in Zn-TFTPA/NH_4_F.

**Figure S36 and S37** show the representative energy-dispersive X-ray (EDX) elemental mapping of our samples Zn-TFTPA, Zn-TFTPA/NH_4_F, indicating the coexistence and uniform distribution of Zn, C, O, and F throughout the samples.


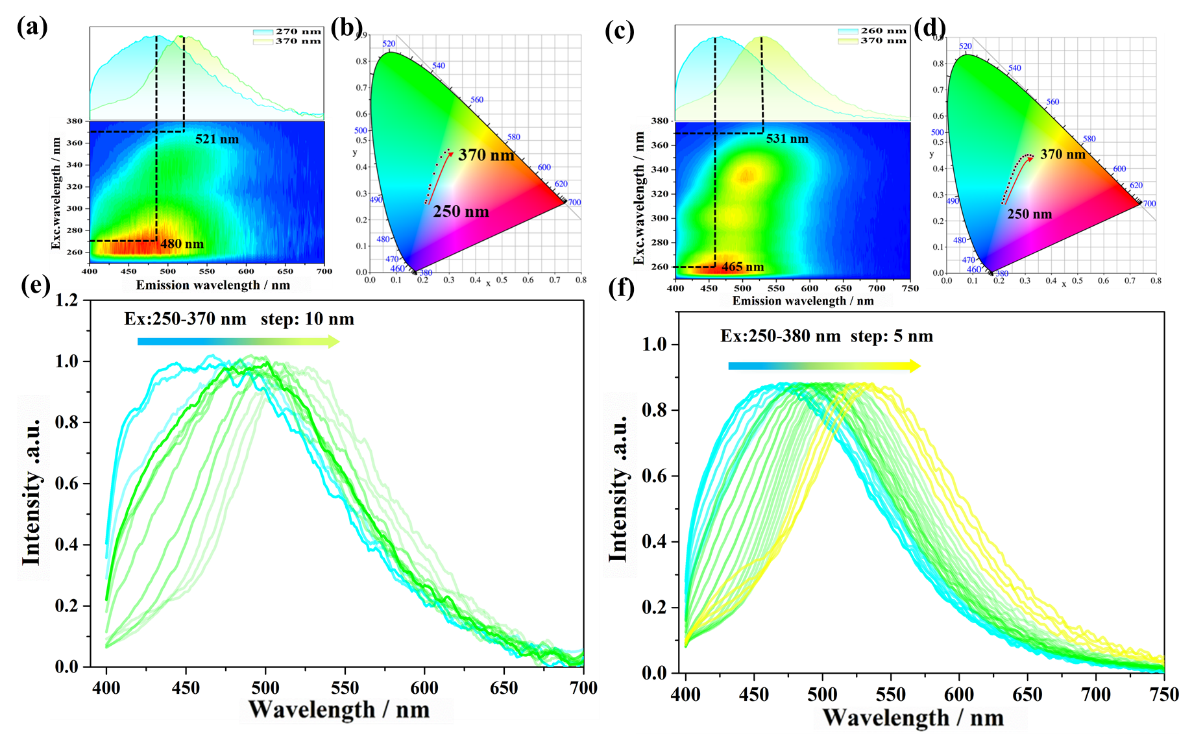


**Figure S38**. Photoluminescence characterization of Zn-TFTPA and Zn-TFTPA/NH_4_F powder under ambient conditions. a) Excitation–phosphorescence mapping of Zn-TFTPA powder under ambient conditions. The upper inset shows the UOP spectra of the Zn-TFTPA powder under the excitation at 270 nm (cyan) and 370 nm (green), respectively. c) Excitation–phosphorescence mapping of Zn-TFTPA/NH_4_F powder under ambient conditions. The upper inset shows the UOP spectra of the Zn-TFTPA/NH_4_F powder following excitation at 260 nm (cyan) and 370 nm (green) respectively. b,d) CIE coordinate diagram of Zn-TFTPA and Zn-TFTPA/NH_4_F by changing the excitation wavelengths. e,f) Excitation dependent phosphorescence spectras of Zn-TFTPA and Zn-TFTPA/NH_4_F.


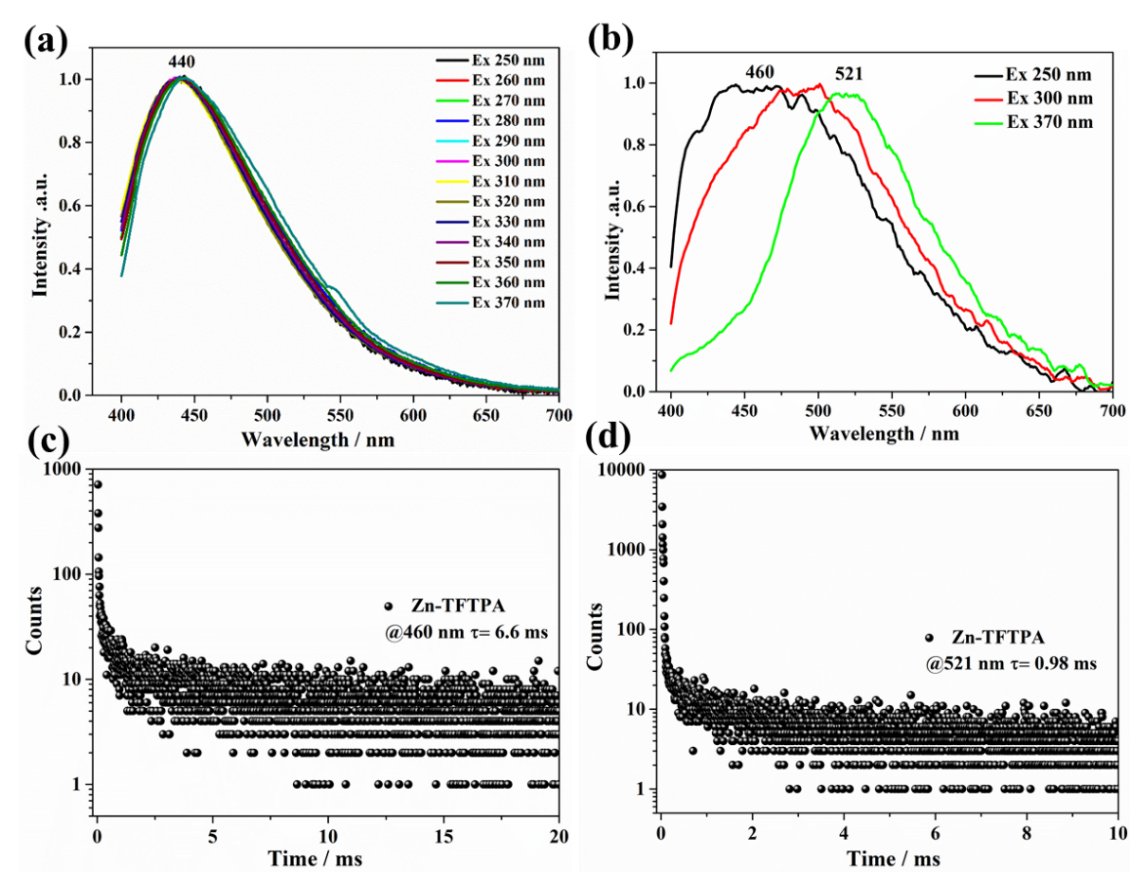


**Figure S39.** Fluorescence spectra (a), delayed PL spectra (b) and decay curves (c, d) of Zn-TFTPA at 460 nm and 522 nm.


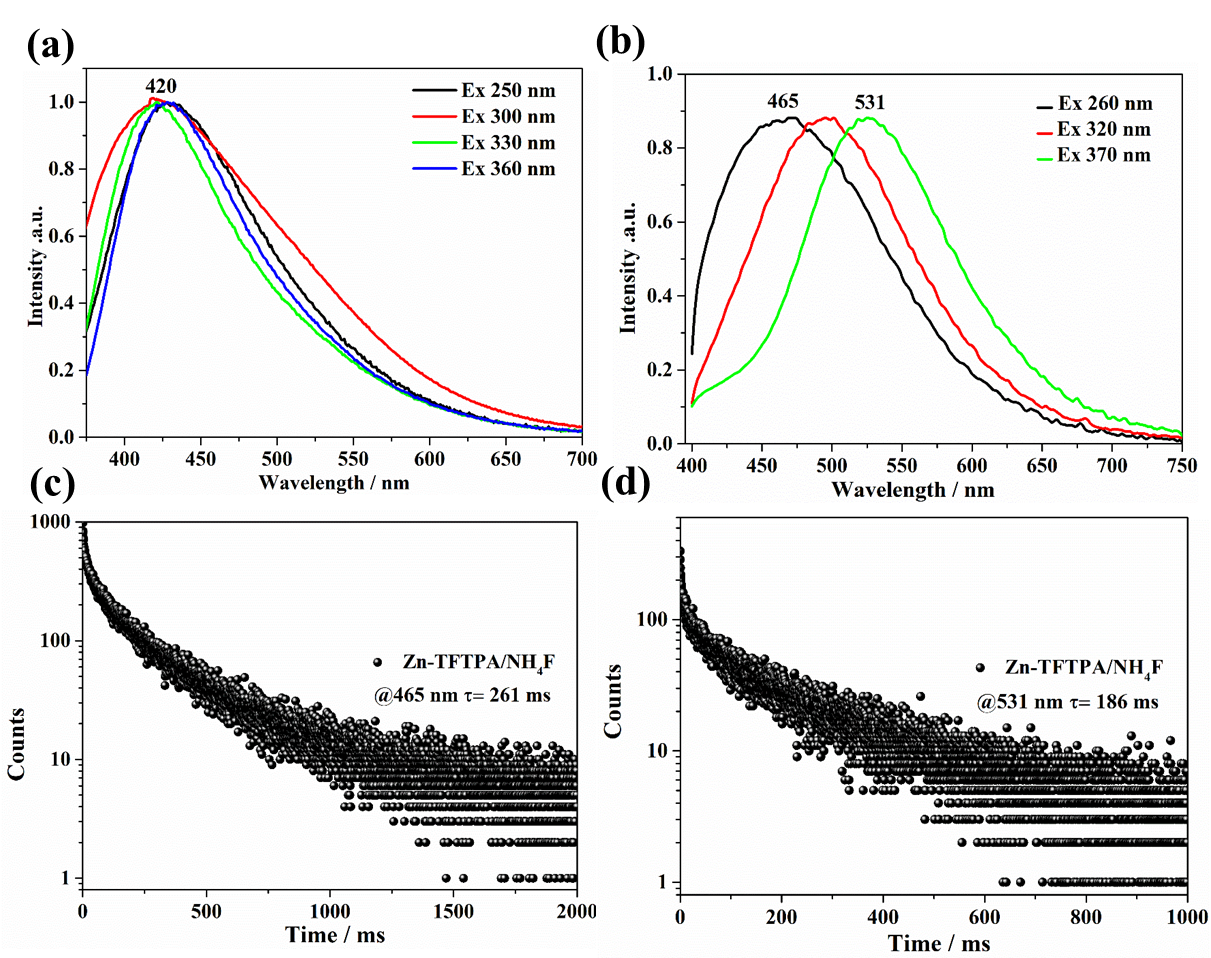


**Figure S40.** Fluorescence spectra (a), delayed PL spectra (b) and decay curves (c, d) of Zn-TFTPA/NH_4_F at 465 nm and 530 nm.

**Table** **S1** Phosphorescence lifetimes (τ) of Cd-TFTPA, Cd-TFTPA/NH_4_F, Cd/Mn-TFTPA/NH_4_F, Cd/Pb-TFTPA/NH_4_F, Zn-TFTPA, Zn-TFTPA/NH_4_F.

| Component | Wavelength (nm) | τ_1_ (ms) | A_1_ (%) | τ_2_ (ms) | A_2_ (%) | τ_3_ (ms) | A_3_ (%) | <τ> (ms) | χ^2^ |
| --- | --- | --- | --- | --- | --- | --- | --- | --- | --- |
| Cd-TFTPA | 445  533 | 19.73  5.87 | 7.40  7.72 | 148.67  48.19 | 42.31  41.10 | 455.57  170.68 | 50.29  51.18 | 290.38  107.43 | 1.145  1.235 |
| Cd-TFTPA/NH_4_F | 417  533 | 56.00  3.19 | 7.26  2.14 | 225.26  51.52 | 45.56  25.57 | 544.91  180.60 | 47.18  72.29 | 363.33  142.12 | 1.170  1.172 |
| Cd/Mn-TFTPA/NH_4_F | 417  520  614 | 16.82  97.03  9.87 | 8.57  82.37  28.39 | 122.92  9.36  33.84 | 39.05  17.63  60.89 | 411.50  108.08 | 52.39  10.72 | 265.35  81.57  35.08 | 1.241  1.121  1.151 |
| Cd/Pb-TFTPA/NH_4_F | 435  550 | 1.45  0.20 | 30.26  32.16 | 9.96  15.89 | 29.26  67.84 | 103.37 | 4048 | 45.04  10.47 | 1.264  1.030 |
| Zn-TFTPA | 460  522 | 0.041  0.011 | 4.11  35.21 | 0.49  0.14 | 13.86  11.55 | 7.92  1.80 | 82.03  53.25 | 6.56  0.98 | 1.295  1.297 |
| Zn-TFTPA/NH_4_F | 465  530 | 12.64  18.48 | 5.45  12.13 | 104.40  208.93 | 32.97  87.87 | 366.46 | 61.58 | 260.7  185.89 | 1.156  1.274 |

<τ>=∑A_j_τ_j_^2^/∑A_j_τ_j_, j=1,2,3...
